# Comparative profiles of anomalous self-experiences and social cognition in clinical high risk for psychosis and autism spectrum disorder

**DOI:** 10.1016/j.scog.2026.100450

**Published:** 2026-06-11

**Authors:** Kristina Ballestad Gundersen, Tina Dam Kristensen, Alberte C.E. Jeppesen, Johannes Andresen, Helle Gade Andersen, Birgitte Fagerlund, Paolo Fusar-Poli, Merete Nordentoft, Bjørn H. Ebdrup, Scott W. Woods, Martha E. Shenton, Barnaby Nelson, Accelerating Medicines Partnership® Schizophrenia (AMP® SCZ), Louise Birkedal Glenthøj

**Affiliations:** a VIRTU Research Group, Mental Health Center Copenhagen, Copenhagen University Hospital - Bispebjerg and Frederiksberg, Copenhagen, Denmark; b Center for Neuropsychiatric Schizophrenia Research, Mental Health Centre Glostrup, Copenhagen University Hospital - Amager and Hvidovre, Denmark; c Department of Psychology, University of Copenhagen, Copenhagen, Denmark; d Department of Clinical Medicine, Faculty of Health and Medical Sciences, University of Copenhagen, Copenhagen, Denmark; e Orygen, The National Centre of Excellence in Youth Mental Health, Australia; f Centre for Youth Mental Health, University of Melbourne, Melbourne, VIC, Australia; g CORE- Copenhagen Centre for Mental Health Research, Bispebjerg Frederiksberg Hospital, Copenhagen, Denmark; h Department of Brain and Behavioral Sciences, University of Pavia, Italy; i Early Psychosis: Interventions and Clinical-detection (EPIC) Lab, Department of Psychosis Studies, King's College London, UK; j Outreach and Support in South-London (OASIS) service, South London and Maudlsey (SLaM) NHS Foundation Trust, UK; k Child and Adolescent Mental Health Center, Copenhagen University Hospital - Bispebjerg Frederiksberg Hospital, Copenhagen, Denmark; l Department of Psychiatry, Yale University School of Medicine, New Haven, CT, USA; m Department of Radiology, Brigham and Women's Hospital and Harvard Medical School, Boston, MA, USA; n Department of Psychiatry, Massachusetts General Hospital and Harvard Medical School, Boston, MA, USA; o Department of Psychiatry, Brigham and Women's Hospital and Harvard Medical School, Boston, MA, USA

**Keywords:** Social cognition, Anomalous self-experience, Ultra high risk, Autism spectrum disorder, Social cognitive bias, Social functioning

## Abstract

**Background:**

Self-disorders, or anomalous disturbances in the basic sense of self, have been described in psychosis-risk states and may contribute to social cognitive impairment and functioning. This study examined self-disorder severity and associations with social cognition and functioning across individuals at clinical high risk for psychosis (CHR—P), autism spectrum disorder (ASD), and controls. ASD was included due to overlapping difficulties in social cognition/functioning with CHR—P. We hypothesized a graded pattern of self-disorders (CHR-P > ASD > controls) and stronger associations between self-disorders and social cognition in CHR—P.

**Methods:**

We included 39 CHR—P, 39 ASD, and 30 controls (mean age ≈24 years; 51% female). Self-disorders were assessed with the Inventory of Psychotic-Like Anomalous Self-Experiences (IPASE). Social cognitive domains included social cognitive bias and theory of mind alongside measures of social functioning. Analyses of covariance and bootstrapped regression models with IPASE as predictor were used, adjusting for age, sex, and IQ. Multiple comparisons were Benjamini-Hochberg corrected.

**Results:**

Self-disorders followed a graded distribution (CHR-P > ASD > controls) across all IPASE subscores. Both clinical groups showed elevated social cognitive bias and reduced social functioning compared to controls. Self-disorders were associated with social cognitive bias in ASD (B = 0.392, 95%CI 0.236–0.544, *p* < 0.001), but not in CHR P. Group differences in associations between self-disorders and social cognition/functioning did not survive correction for multiple comparisons.

**Conclusion:**

Self-disorders showed a graded distribution across groups but was only associated with social cognitive deficits in ASD. Findings suggest that shared symptom profiles may reflect divergent experiential processes across groups. Further research is needed to clarify clinical implications and generalizability.

## Highlights

• Self-disturbances were linked to social cognitive bias only in the autism group.
• Self-disturbances were highest in psychosis-risk, intermediate in autism and lowest in controls.
• Findings suggest distinct self-experience profiles across clinical populations.

## 1 Introduction

Disturbances in self-experience have been described in schizophrenia-spectrum psychopathology and have also been reported in individuals at clinical high risk for psychosis (CHR—P) (Sass and Parnas, 2003; Raballo et al., 2021; Raballo et al., 2016). These disturbances, often referred to as anomalous self-experiences (ASEs), disturbances of ipseity or self-disorders, involve alterations in the basic first-person sense of self: the immediate sense that one's thoughts, experiences, and perspective belong to oneself (Sass and Parnas, 2003). Within phenomenological models of psychosis vulnerability, ASEs include hyperreflexivity, or heightened self-consciousness; altered self-world boundaries, such as reduced felt distance between self and world/others; and diminished self-presence, a weakened, normally taken-for-granted sense that one's experiences are one's own (Sass and Parnas, 2003).

These alterations may be important for understanding social cognition and functioning (Nelson et al., 2009). Social interaction relies not only on explicit cognitive skills (e.g., inferring beliefs), but also on implicit capacities for distinguishing self from other, and participating in shared social situations. Phenomenological accounts propose that disturbances in the basic sense of self may interfere with implicit social processes, potentially affecting how individuals perceive, interpret, and respond to others (Nelson et al., 2009). Accordingly, ASEs have been hypothesized to contribute to social cognitive and functional difficulties in schizophrenia spectrum disorders (SSD) (Nelson et al., 2009). Empirical studies have reported associations between ASEs and impaired global functioning (Comparelli et al., 2016; Haug et al., 2014), and with deficits in emotion processing and social perception (Cicero et al., 2016; Ebisch et al., 2013; Henriksen et al., 2021). However, evidence linking ASEs specifically to performance-based social capacity or community functioning remains limited, particularly in CHR-P and in comparison with other clinical conditions characterized by social difficulties.

Autism spectrum disorder (ASD) provides a clinically relevant comparator because ASD and SSD are both associated with marked impairments in social cognition and functioning, contributing to overlapping clinical presentations and diagnostic complexity (Oliver et al., 2021; Sasson et al., 2011). This overlap may be especially relevant in early psychosis-risk presentations, where positive symptoms may be subtle or nonspecific and social difficulties more prominent (Vaquerizo-Serrano et al., 2022). Social cognition includes domains such as emotion processing, theory of mind (ToM), attributional style, and social perception (Pinkham et al., 2014), whereas social functioning refers to the ability to navigate social environments, maintain relationships, and fulfill social roles. Both CHR-P and ASD exhibit difficulties across these domains (Barbato et al., 2015; Piskulic et al., 2016; Addington et al., 2008; Pinkham et al., 2020; Scheeren et al., 2022; Velikonja et al., 2019), although direct comparisons have yielded mixed results, with some studies reporting distinct domain-specific profiles (Pinkham et al., 2020; Rashidi et al., 2025), and others suggesting broader overlap (Oliver et al., 2021; Ozbek et al., 2023).

Beyond shared social difficulties, CHR-P and ASD may differ in how altered self-experience is organized and clinically expressed. In ASD, altered self-experiences are generally conceptualized in relation to self-other understanding, perspective-taking, and intersubjective engagement, rather than primary ipseity disturbance characteristic of SSD (Nilsson et al., 2019; Jeličić et al., 2025). If ASE-like experiences in CHR-P and ASD reflect different underlying organizations of self- and other-experience, they may also show different associations with social cognition and functioning. This comparison may help clarify whether potential ASE-related social difficulties are specific to CHR-P or also observed in ASD. However, to date, no studies have directly compared these associations across CHR-P and ASD populations.

ASEs are commonly assessed with the clinician-administered Examination of Anomalous Self Experience (EASE; (Parnas et al., 2005)), whereas the Inventory of Psychotic Like Anomalous Self Experiences (IPASE) is a validated self-report questionnaire proposed for more feasible screening (Cicero et al., 2017; Nelson et al., 2019; Magnani et al., 2023). However, the IPASE has not been applied in ASD nor directly compared across CHR—P, ASD, and controls.

The aim of this study was to investigate ASE severity and its associations with social cognition and social functioning, operationalized using both functional capacity measures and real-world community functioning, across CHR—P, ASD, and CC groups. Based on evidence that self-disturbances are strongly associated with SSD and CHR-P (Sass and Parnas, 2003; Raballo et al., 2021; Raballo et al., 2016), but that altered self-experience may also occur in ASD in a phenomenologically distinguishable form (Nilsson et al., 2019), we hypothesized a graded pattern of ASE severity (CHR-P > ASD > controls). We further hypothesized that ASEs would be more strongly associated with social cognitive difficulties in CHR-P compared to ASD or controls.

## 2 Methods

### 2.1 Participants

Participants were drawn from two independent cohorts in the Copenhagen area: 39 CHR-P individuals from the Danish site of the PRESCIENT trial within the Accelerating Medicines Partnership® Schizophrenia (AMP® SCZ) consortium (Wannan et al., 2024), and 39 individuals with ASD from the randomized clinical trial STEPS (Andresen et al., 2025). Thirty controls were included, comprising 15 from PRESCIENT and 15 from EYEdentify, a STEPS subproject (Jeppesen et al., 2025).

CHR-P and ASD participants were recruited through psychiatric services, whereas controls were recruited from the general population. Eligibility and screening followed the originating study protocols, with additional harmonization for cross-cohort analyses (full eligibility criteria and screening instruments in Appendix A (Tables A.1-A.2). Inclusion was restricted to participants aged 18–30 years, with estimated IQ > 70 and no self-reported organic brain disease (ICD-10: F00–F09) or central nervous system disease. Medication use was recorded by self-report and verified in clinical records when available.

In PRESCIENT, CHR-P status was determined using the Positive SYmptoms and Diagnostic Criteria for the CAARMS Harmonized with the SIPS (PSYCHS), which operationalizes risk syndromes including Attenuated Positive Symptom Syndrome, Brief Intermittent Psychotic Syndrome, and Genetic Risk with Functional Decline (Woods et al., 2024). CHR-P participants were antipsychotic-free (i.e., equivalent to a total lifetime haloperidol dose of <50 mg) and excluded for current or past psychotic disorder. Controls from PRESCIENT were classified as community controls (CC). A psychiatric diagnosis apart from within the psychosis spectrum was accepted and they were excluded if they met CHR-P criteria, had a current or past Cluster A personality disorder, used psychotropic medication, or had a first-degree family history of SSD. In EYEdentify, controls were healthy controls, excluded for any current psychiatric disorder, including substance use disorders (Jeppesen et al., 2025). Throughout the manuscript, the pooled control group is referred to as CC.

ASD participants had a documented pre-existing ASD diagnosis (ICD-10: F84.0, F84.1, F84.5, F84.8) based on autism-specific instruments (e.g., Adult Asperger Assessment (Baron-Cohen et al., 2005); Autism Diagnostic Observation Schedule, Second Edition (Lord, 2012); and Autism Diagnostic Interview-Revised (Rutter et al., 2003)) and current social impairment, defined by a Social Responsiveness Scale self-report T-score ≥ 60 (Bruni, 2014).

The study was conducted in accordance with the Declaration of Helsinki. Both studies were approved by the Regional Committee on Health Research Ethics of the Capital Region of Denmark (PRESCIENT: H-21033096; STEPS: H-23055504) and the Danish Data Protection Agency (PRESCIENT: UO1MH124631–6; STEPS: P-2023-14,488). All participants provided written informed consent.

### 2.2 Measures

All measures used in this study were collected at baseline from the respective original studies. Clinical interviews were conducted by trained clinical psychologists or medical doctors. Social cognitive tests were administered by clinical psychologists or supervised clinical psychology students.

#### 2.2.1 Clinical diagnoses and symptom assessment

CHR-P symptoms were evaluated using the PSYCHS instrument, which captures dimensional ratings of attenuated and brief intermittent psychotic symptoms (Woods et al., 2024). In PRESCIENT, Cluster A personality disorders were assessed using the Structured Clinical Interview for DSM-5 Personality Disorders (First et al., 2016), and first-degree family history of SSD was assessed using the family interview for genetic studies. In STEPS/EYEdentify, psychiatric diagnoses were assessed with the Mini-International Neuropsychiatric Interview (Sheehan et al., 1998).

#### 2.2.2 Anomalous self-experience measure

ASEs were assessed using IPASE, a 57-item self-report questionnaire rated on a 5-point Likert scale (Cicero et al., 2017). The IPASE yields a total score and five subscales: cognition, reflecting disturbances in thinking and attention; self-awareness and presence, reflecting altered self-presence and first-person perspective; consciousness, reflecting changes in the flow or quality of conscious experience; somatization, reflecting anomalous bodily experiences; and demarcation/transitivism, reflecting altered boundaries between self and others/world. The IPASE shows strong convergent validity with clinician-rated ASEs assessed using EASE, with reported Pearson *r* = 0.92 between total scores (Nelson et al., 2019).

#### 2.2.3 Social cognition and functioning measures

Key domains of social cognition and functioning were assessed using validated instruments selected a priori for their theoretical relevance to CHR-P and ASD populations (Pinkham et al., 2014; Sasson et al., 2020). ToM was assessed using The Awareness of Social Inference Test - short (TASIT-S), which also captures aspects of social perception (McDonald et al., 2006; Honan et al., 2016). Social cognitive bias was measured with the Davos Assessment of Cognitive Biases Scale (DACOBS), a self-report questionnaire assessing cognitive biases relevant to social interpretation, such as jumping to conclusions, belief inflexibility, attention to threat, and external attribution (van der Gaag et al., 2013). Social functioning was assessed using the Social Skills Performance Assessment (SSPA), a performance-based role-play measure of functional capacity (Patterson et al., 2001), and the Personal and Social Performance Scale (PSP), a clinician-rated semi-structured interview of real-world community functioning (White et al., 2016). SSPA role-plays were audio-recorded and scored by trained raters. PSP ratings were based on participant interview only, without collateral informant information. Estimated IQ was derived from the Matrix Reasoning and Vocabulary subtests of the Danish Wechsler Adult Intelligence Scale - fourth edition (WAIS-IV), found to be strongly correlated with full-scale IQ (Axelrod, 2002).

### 2.3 Statistical analyses

All analyses were conducted in IBM SPSS v28, with visualizations in R v4.5.1. Inspection of variable distributions indicated marked non-normality, primarily due to floor and ceiling effects in CCs, and to a lesser extent, the ASD group. Accordingly, nonparametric methods and bootstrapped estimates were applied when appropriate.

Group differences in IPASE, social cognition, and social functioning were analyzed using ANCOVA, adjusting for age, sex assigned at birth, and estimated IQ. IQ was included as covariate to control for general cognitive ability that may covary with task performance and functioning (Green et al., 2000). False discovery rate (FDR) correction (Benjamini–Hochberg, q = 0.05) was applied within conceptually defined families of hypotheses. Model residuals were visually inspected and met assumptions of normality and homoscedasticity.

Associations between ASEs and social cognitive/ functional variables were examined using Spearman rank correlations (ρ) within each group, with FDR correction applied within each variable domain. Exploratory subgroup analysis within the ASD group used a cutoff of IPASE ≥104 (minimum CHR-P score) to examine within-group variation.

To test associations and group differences in ASE-variable relationships, hierarchical linear regression models were fit for each variable domain (DACOBS, SSPA, PSP, TASIT-S). Models included covariates (age, sex, IQ), main effects (IPASE total or subscale score; groups), and interaction terms (IPASE × group). Group × ASE interaction terms were included to test whether ASE-variable associations differed across groups. Bootstrap resampling (5000 bias-corrected and accelerated resamples, stratified by group) provided confidence intervals and robustness checks. Regression diagnostics included checks for normality, multicollinearity, and influential observations. FDR correction was applied across interaction terms and within-group slope estimates.

Missing data were minimal (DACOBS, 1; SSPA, 1; estimated IQ, 7). Primary analyses used complete-case models (listwise deletion). Multiple imputation was not applied due to SPSS limitations with pooled inference in ANCOVA and incompatibility with bootstrapped regression procedures. Sensitivity analyses were conducted to evaluate the robustness of findings, including models excluding IQ, or all covariates. Given the higher prevalence of stimulant use in the ASD group, additional sensitivity analyses included stimulant use as a covariate. Model diagnostics and robustness checks were conducted and are reported in full in Appendix B.

## 3 Results

### 3.1 Participant characteristics

Demographic and clinical characteristics are summarized in Table 1. Groups did not differ significantly in age, sex assigned at birth, income classification, or participant and parental education. Estimated IQ was significantly higher in the ASD group with no difference between CHR-P and CC. Among clinical groups, medication use differed, with higher rates of stimulant and overall medication use in the ASD group. No other medication categories showed significant group differences.

**Table 1:** Sociodemographic and clinical data on all participants.

Variables	CHR-P	ASD	CC		
	(N = 39)	(*N* = 39)	(*N* = 30)	F-value / χ^2^	*p*
Age, mean (SD)	24 (4)	25 (3)	24 (2)	F = 0.056	*p* = 0.946
Female, n (%)	22 (56%)	17 (44%)	16 (53%)		*p* = 0.502
PSYCHS syndrome n (%) a					
APSS	39 (100%)	–	–	–	
BIPS	2 (5%)	–	–	–	
GRD	5 (13%)	–	–	–	
Medication use, n (%) b					
No medication	23 (59%)	13 (33%)	–	χ^2^ = 5.159	*p* = 0.023
Antipsychotics	0 (0%)	1 (3%)	–	χ^2^ = 1.013	*p* = 0.314
Antidepressants	9 (23%)	16 (41%)	–	χ^2^ = 2.885	*p* = 0.089
Anxiolytics	3 (8%)	2 (5%)	–	χ^2^ = 0.214	*p* = 0.644
Stimulants	4 (10%)	17 (44%)	–	χ^2^ = 11.013	*p* < 0.001
Hypnotics	1 (3%)	0 (0%)	–	χ^2^ = 1.013	*p* = 0.314
Income level, n (%)				χ^2^ = 3.605	*p* = 0.165
High income country	37 (95%)	39 (100%)	30 (100%)		
Upper middle-income country	2 (5%)	0 (0%)	0 (0%)		
Level of education, n (%)				χ^2^ = 6.526	*p* = 0.367
Junior high school	7 (18%)	8 (21%)	2 (7%)		
Completed high school	23 (59%)	23 (59%)	17 (57%)		
Bachelor's degree or equivalent	9 (23%)	6 (15%)	10 (33%)		
Master's degree or equivalent	0 (0%)	2 (5%)	1 (3%)		
Parental level of education, n (%)				χ^2^ = 6.607	*p* = 0.580
Less than junior high school	2 (5%)	0 (0%)	0 (0%)		
Junior high school	1 (3%)	1 (3%)	0 (0%)		
Completed high school	4 (11%)	7 (18%)	5 (17%)		
Bachelor's degree or equivalent	22 (58%)	20 (51%)	14 (47%)		
Master's degree or equivalent	9 (24%)	11 (28%)	11 (37%)		
Estimated IQ, mean (SD)	107 (11) (*n* = 35)	117 (9)	105 (11) (*n* = 27)	F = 14.291	*p* < 0.001

***Note.*** Group differences were assessed using one-way ANOVA for continuous variables and Pearson's chi-square tests for categorical variables. CHR—P, clinical high risk for psychosis; ASD, autism spectrum disorder; CC, community controls; SD, standard deviation; PSYCHS, Positive SYmptoms and Diagnostic Criteria for the CAARMS Harmonized with the SIPS; APSS, Attenuated Positive Symptom Syndrome; BIPS, Brief Intermittent Psychotic Syndrome; GRD, Genetic Risk with Functional Decline; IQ, intelligence quotient.

a All CHR-P participants met criteria for APSS, 2 also met BIPS criteria and 5 met GRD criteria.

b Comparisons were conducted between CHR-P and ASD groups only as psychotropic medication use being exclusion criteria in CC. Medication use categories are not mutually exclusive. One CHR-P and eleven ASD participants used two or more psychotropic medications.

### 3.2 Group differences in anomalous self-experiences

**Fig. 1:**
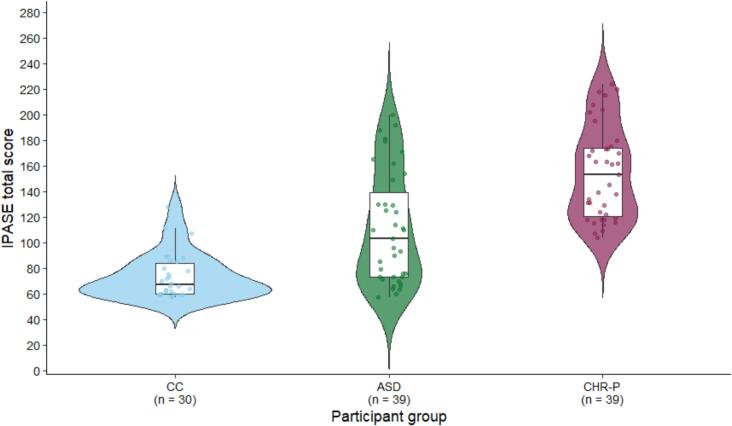
IPASE total scores by group: raw distributions. ***Note***. Figure shows raw IPASE total scores by group (violin plots with individual data points and embedded boxplots). Group sample sizes are shown on the x-axis. CHR—P, clinical high risk for psychosis; ASD, autism spectrum disorder; CC, community controls; IPASE, the inventory for psychotic like anomalous self-experiences.

A significant group effect was observed on IPASE total scores (adjusted F(2,95) = 42.52, *p* < 0.001, η^2^ = 0.472), with a graded pattern: CHR-P > ASD > CC (Table 2). Pairwise comparisons confirmed significantly higher IPASE total scores in CHR-P than ASD (ΔM = 42.59, *p* < 0.001) and CC (ΔM = 84.19, *p* < 0.001), and in ASD than CC (ΔM = 41.61, *p* < 0.001), after covariate adjustment and FDR correction. The raw score distribution by group is visualized in Fig. 1. Subscale analyses showed significant group effects across all domains (all *p* < 0.001), following the same graded pattern. Detailed pairwise comparisons and unadjusted model statistics are reported in Appendix C (Tables C.1-C.2).

**Table 2:** Group differences in IPASE and social cognition scores (ANCOVA adjusted for age, sex assigned at birth and estimated IQ).

	CHR-P	ASD	CC			
Variables	Mean (SD)	Mean (SD)	Mean (SD)	F (df_1_, df_2_), *p*	Partial η^2^	Post-hoc FDR corrected (ΔM)
IPASE						
Total score	154.6 (36.4)	110.9 (43.7)	73.8 (17.7)	F (2, 95) = 42.515, ***p* < 0.001**⁎	0.472	CHR-P > ASD > CC
Cognition	14.2 (4.9)	10.7 (4.5)	8.5 (2.4)	F (2, 95) = 14.961, ***p* < 0.001**⁎	0.240	CHR-P > ASD > CC
Self-awareness and presence	64.5 (16.5)	43.0 (19.9)	27.5 (7.3)	F (2, 95) = 46.242, ***p* < 0.001**⁎	0.493	CHR-P > ASD > CC
Consciousness	19.2 (5.8)	14.8 (6.7	9.2 (3.3)	F (2, 95) = 26.501, ***p* < 0.001**⁎	0.358	CHR-P > ASD > CC
Somatization	43.9 (11.4)	33.3 (13.4)	22.4 (6.2)	F (2, 95) = 30.091, ***p* < 0.001**⁎	0.388	CHR-P > ASD > CC
Demarcation/ Transitivism	12.82 (3.9)	9.1 (3.6)	6.2 (1.5)	F (2, 95) = 32.571, ***p* < 0.001**⁎	0.407	CHR-P > ASD > CC
DACOBS	150.1 (23.7)	147.1 (26.8)	104.1 (19.7)	F (2. 94) = 34.541, ***p* < 0.001**⁎	0.424	CHR-P/ASD > CC
TASIT-S	28.9 (3.5)	29.2 (3.2)	29.8 (3.4)	F (2, 95) = 0.799, *p = 0*.453	0.017	CHR-P/ASD/CC
SSPA	66.6 (8.4)	59.7 (9.1)	74.6 (3.9)	F (2, 94) = 27.849, ***p* < 0.001**⁎	0.372	CC > CHR-P > ASD
PSP	54.2 (11.7)	54.2 (17.6)	89.1 (6.5)	F (2, 95) = 62.042, ***p* < 0.001**⁎	0.566	CC > CHR-P/ASD

***Note.*** Unadjusted means (SD) are shown. ANCOVAs adjusted for age, estimated IQ, and sex assigned at birth. Pairwise comparisons were conducted using estimated marginal means. For post hoc pairwise comparisons, “>” indicates a statistically higher score (*p* < 0.05). “/” indicates no significant difference. CHR—P, clinical high risk for psychosis; ASD, autism spectrum disorder; CC, community controls; SD, standard deviation; IPASE, the inventory for psychotic like anomalous self experiences; DACOBS, davos assessment of cognitive biases scale; TASIT-S, the awareness of social inference test - short form; PSP, personal and social performance scale; SSPA, social skills performance assessment.

⁎ Bold values indicate statistical significance after Benjamini-Hochberg false discovery rate correction for multiple testing.

### 3.3 Group differences in social cognition and functioning

Social cognitive bias, measured by DACOBS, differed significantly by group (*F*(2,94) = 34.54, *p* < 0.001, η^2^ = 0.424), with both CHR-P and ASD scoring higher than controls (*p*s < 0.001), and no difference between clinical groups. Similar group differences were observed in social functioning. Significant group differences were observed for PSP (*F*(2,95) = 62.04, *p* < 0.001, η^2^ = 0.566) and SSPA (*F*(2,94) = 27.85, *p* < 0.001, η^2^ = 0.372), with CHR-P and ASD scoring lower than controls (*p*s < 0.001). On SSPA, ASD participants scored lower than CHR-P (*p* < 0.001). No group differences were observed for TASIT-S (*F*(2,95) = 0.80, *p* = 0.453). All results are summarized in Table 2. Detailed pairwise comparisons and unadjusted model statistics are reported in Appendix C (Tables C.1-C.2).

### 3.4 Associations between anomalous self-experiences and social cognition and functioning

Regression results examining associations between IPASE total scores and social cognitive and functional variables across groups are summarized in Table 3. In the ASD group, higher IPASE scores were significantly associated with increased social cognitive bias (DACOBS; B = 0.392, *p* < 0.001) and poorer real-world community functioning (PSP; B = −0.143, *p* = 0.029), though only the DACOBS association remained significant after correction. No significant interactions emerged at the total score level. Exploratory sensitivity analyses suggested a nominally stronger ASE - real-world community functioning link in ASD compared to CHR-P (Appendix B, Table B.5).

**Table 3:** Bootstrapped hierarchical linear regression models examining associations between IPASE and social cognitive and functional variables, moderated by group.

		Conditional slope of IPASE, B [95% CI], *p*			ΔSlope vs CHR—P, B [95% CI], *p*			
Variable	N	CHR-P	ASD	CC	ASD – CHR-P	CC – CHR-P	R^2^ (full model)	ΔR^2^ (interaction)
DACOBS	100	0.199 (−0.030, 0.413), *p* = 0.091	0.392 (0.236, 0.544), *p* < **0.001**⁎	0.324 (−0.217, 0.756*), p* = 0.122	0.193 (−0.084, 0.478), *p* = 0.162	0.125 (−0.441, 0.649), *p* = 0.598	0.562	0.010
TASIT-S	101	0.002 (−0.038, 0.037), *p* = 0.914	0.001 (−0.026, 0.032), *p* = 0.932	−0.013 (−0.135, 0.055), *p* = 0.743	0.000 (−0.048, 0.050), *p* = 0.985	−0.015 (−0.130, 0.061), *p* = 0.739	0.055	0.001
PSP	101	−0.019 (−0.136, 0.088), *p* = 0.740	−0.143 (−0.261, −0.012), *p* = 0.029	−0.096 (−0.232, 0.026), *p* = 0.108	−0.123 (−0.287, 0.064), *p* = 0.160	−0.077 (−0.261, 0.094), *p* = 0.366	0.627	0.010
SSPA	100	0.022 (−0.065, 0.119), *p* = 0.618	0.006 (−0.051, 0.059), *p* = 0.823	−0.010 (−0.107, 0.092), *p* = 0.796	−0.016 (−0.118, 0.080), *p* = 0.767	−0.032 (−0.161, 0.098), *p* = 0.577	0.393	0.001

***Note.*** Bootstrapped hierarchical linear regression models were estimated separately for each variable (total score) and adjusted for covariates (age, sex, and estimated IQ). Values represent unstandardized regression coefficients (B) with bias-corrected and accelerated 95% confidence intervals (CI) based on 5000 stratified bootstrap resamples. Group-specific slopes (CHR—P, ASD, CC) are conditional effects from the interaction model. R^2^ indicates full model fit. ΔR^2^ reflects the additional variance explained by interaction terms. CHR—P, clinical high risk for psychosis; ASD, autism spectrum disorder; CC, community controls; IPASE, the inventory for psychotic like anomalous self experiences; DACOBS, davos assessment of cognitive biases scale; TASIT-S, the awareness of social inference test - short form; PSP, personal and social performance scale; SSPA, social skills performance assessment.

⁎ Bold values indicate statistical significance after Benjamini-Hochberg false discovery rate correction for multiple testing.

Domain-level models using IPASE subscales indicated that the Cognition subscale was associated with both higher DACOBS scores and lower PSP scores in ASD, with significantly stronger slopes than in CHR-P (DACOBS ASD-CHR-P slope difference: B = 3.197, *p* = 0.017; PSP ASD-CHR-P slope difference: B = −1.967, *p* = 0.010). No subscale interactions survived FDR correction (Appendix B, Table B.7).

Across other variables (SSPA, TASIT-S), no significant associations were observed. Model fit was strongest for PSP (R^2^ = 0.627) and DACOBS (R^2^ = 0.562), and lowest for TASIT-S (R^2^ = 0.055). Interaction blocks explained minimal additional variance (ΔR^2^ < 0.01). Across all sensitivity analyses, results were comparable to the primary analyses, supporting the robustness of the findings. Full model diagnostics and robustness checks are reported in Appendix B (Table B.1-B.7).

### 3.5 Results of exploratory analyses

**Fig. 2:**
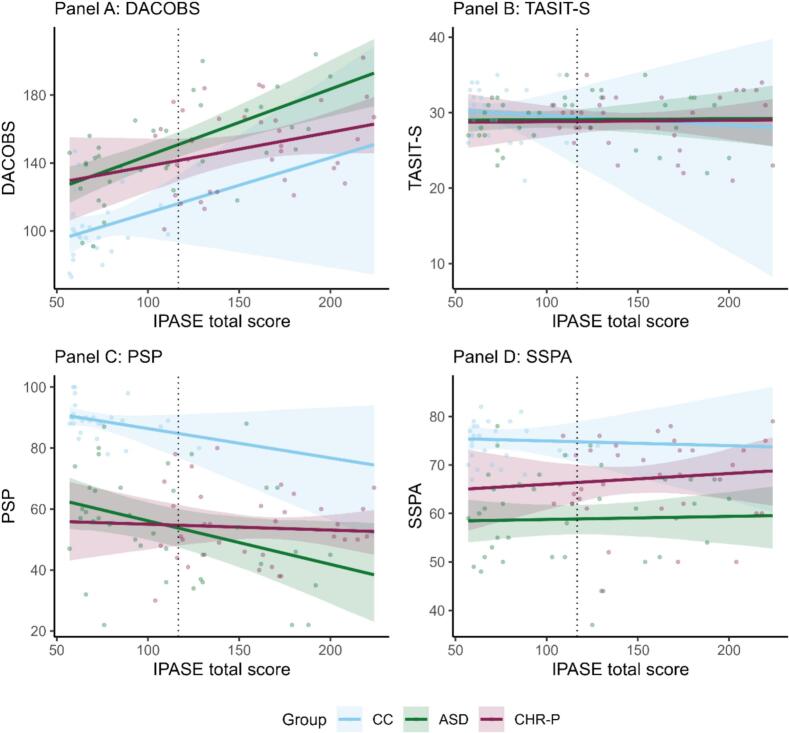
Covariate-adjusted associations between IPASE and social cognition/social functioning variables by group. ***Note.*** Panels show covariate-adjusted, model-estimated variable scores across IPASE by group from an IPASE × group interaction model adjusted for age at baseline, sex assigned at birth, and estimated IQ. Shaded bands indicate bootstrap 95% confidence bands (percentile; 5000 resamples stratified by group; fixed seed). IPASE was centered at the total-sample mean in the model; the dotted vertical line marks this mean, and the x-axis displays raw IPASE scores for ease of interpretation. CHR—P, clinical high risk for psychosis; ASD, autism spectrum disorder; CC, community controls; IPASE, the inventory for psychotic like anomalous self experiences; DACOBS, davos assessment of cognitive biases scale; TASIT-S, the awareness of social inference test - short form; PSP, personal and social performance scale; SSPA, social skills performance assessment.

Visual inspection of interaction plots (Figs. 2–3) and unadjusted scatterplot (Appendix C, Fig. C.3-C.4) suggested stronger ASE-variable associations in ASD, despite nonsignificant interactions. Unadjusted correlations supported the adjusted findings (Appendix D, Fig. D.1), with IPASE-DACOBS associations strongest in ASD (ρ = 0.609, *p* < 0.001). An exploratory analysis within ASD showed that this effect was driven by individuals with high ASE levels (IPASE ≥104: ρ = 0.480, *p* = 0.037; IPASE <104: ρ = 0.186, *p* = 0.447). Associations with PSP were modest in ASD and CC, and null in CHR—P. No significant associations were found for TASIT-S in any group. Within-group correlations between estimated IQ and the social cognitive and functional measures were modest across all groups (|ρ| ≤ 0.28), suggesting that group IQ was unlikely to substantially explain the within-group ASE associations reported above.

## 4 Discussion

This study investigated ASEs, as measured by the IPASE, and their relationship to social cognition and social functioning across CHR—P, ASD and CC groups. Specifically, we compared ASE severity across groups and examined whether associations between ASEs and social cognitive performance and social functioning differed across groups. We hypothesized graded ASE severity (CHR-P > ASD > CC), and stronger associations with social cognition in CHR—P.

### 4.1 Anomalous self-experience severity across groups

Consistent with our severity hypothesis and prior clinician-rated studies of self-disturbance (Nilsson et al., 2020), CHR-P participants exhibited the highest levels of self-disturbance, with ASD participants showing intermediate levels and CC the lowest. This pattern was consistent across all IPASE subscales, supporting a dimensional expression of self-disturbance that spans clinical and non-clinical populations. Notably, this challenges the notion that self-disturbances are specific to the psychosis-spectrum (Parnas and Henriksen, 2014), and supports their broader relevance across neurodevelopmental and clinical risk states.

While the EASE remains the gold standard for in-depth phenomenological assessment of ASEs, its clinical feasibility is limited by resource demands (Cobanovic et al., 2025). In contrast, the IPASE, a self-report measure validated against the EASE (Cicero et al., 2017; Nelson et al., 2019), may offer a scalable tool for initial screening of minimal self-disturbance, albeit one that lacks the phenomenological nuance and contextual sensitivity of clinician-administered interviews. Our findings are the first to demonstrate IPASE group differences in ASD and CHR—P, suggesting potential utility for broader screening of self-disturbance across heterogeneous clinical contexts.

Given the reliance on self-report, it is important to consider potential sources of measurement variability, particularly in ASD. Although the IPASE does not assess psychosis per se, it captures subjective anomalies that phenomenologically overlap with experiences reported in SSD. In ASD, a tendency toward literal or rule-based interpretations (Kalandadze et al., 2018) may affect responses to abstract or ambiguously phrased items (Larson et al., 2017; Wilson et al., 2020). For example, the item “I often look at myself in the mirror to see if I have changed” may be interpreted as referencing physical appearance rather than identity or self-perception. Such divergent interpretations likely reflect distinct experiential frameworks and may be further influenced by alexithymia, which is common in ASD and involves difficulty identifying and describing internal states (Kinnaird et al., 2019). Together, these factors may increase measurement noise and should be considered in future scale refinement.

### 4.2 Anomalous self-experiences and social cognition

Contrary to our expectations, while CHR-P participants showed elevated ASEs, these were not strongly associated with social cognitive domains. In contrast, robust associations between ASEs and social cognition emerged in the ASD group, particularly for social cognitive bias (DACOBS).

These findings diverge from theoretical accounts proposing a close correspondence between minimal self-disturbance and social cognitive deficits particularly in psychosis-risk states (Nelson et al., 2009). While ASEs are salient in CHR—P, they may not align closely with the specific domains of social cognition assessed in this study. This dissociation is noteworthy, as ASEs in CHR-P have been associated with negative and disorganization symptoms, which is clinically relevant given that the diagnostic ambiguity between CHR-P and ASD often arises when social difficulties are prominent but positive symptoms are subtle (Værnes et al., 2019).

In ASD, stronger associations between ASEs and social cognitive bias may indicate that self-disturbances are more closely related to the interpretation of social ambiguity. Prior research suggests that difficulties in disambiguating social cues, particularly under conditions of negative affect or interpersonal stress, can amplify hostile attribution biases in autism (Spain et al., 2016). These findings suggest that similar ASE scores may reflect different underlying experiences across populations, underscoring the importance of considering clinical context when interpreting self-disturbance measures in transdiagnostic research. However, this interpretation should be made cautiously, as both IPASE and DACOBS are self-report measures. Thus, their association may partly reflect shared self-report variance, including participants general response style, current distress, or subjective appraisal of social difficulties.

### 4.3 Shared and distinct social cognitive profiles

Both clinical groups exhibited elevated self-reported social cognitive bias relative to controls, consistent with some literature (An et al., 2010; Morel-Kohlmeyer et al., 2021), although evidence is mixed (Glenthøj et al., 2016; Didehbani et al., 2012). The absence of significant differences between CHR-P and ASD on the DACOBS suggests comparable levels of cognitive biases in social contexts. However, these similarities may reflect distinct underlying patterns, as indicated by our finding that DACOBS scores were associated with ASEs in ASD but not in CHR—P.

No group differences were observed in ToM as measured by the TASIT-S. This contrasts with studies showing ToM impairments in both CHR-P and ASD relative to healthy controls (Pinkham et al., 2020; Glenthøj et al., 2016), and may reflect measurement limitations of the abbreviated TASIT-S or normative variation within the CC group.

### 4.4 Anomalous self-experiences and social functioning

Our study included measures of real-world functioning (PSP) and functional capacity (SSPA), finding differential aspects of social impairment across groups. ASD participants showed the greatest deficits in functional capacity, while both CHR-P and ASD groups exhibited reduced real-world social functioning compared to controls, consistent with evidence that ASD involves enduring social-communication difficulties and context-sensitive constraints on participation (Recio et al., 2024; Bishop-Fitzpatrick et al., 2015). Functional capacity limitations may be more pronounced in ASD than in at-risk mental states, where functional difficulties may vary with symptom fluctuations and phase of illness (Allswede et al., 2020). Future studies should examine whether the comparatively lower functional capacity observed in ASD also extends to comparisons with established psychotic disorders.

Our additional analyses indicated limited overlap between social skills capacity and everyday functioning which aligns with prior findings that real-world functioning depends not only on social competence, but also on motivational factors (e.g., amotivation), contextual demands (e.g., unpredictability), and environmental fit (e.g., sensory load, social support), all of which may vary across clinical groups (Orsmond et al., 2013; Devoe et al., 2020).

Total ASE level was not strongly associated with real-world functional performance or functional capacity across groups. While prior CHR-P studies using the clinician-rated EASE have reported associations between ASEs and the Global Assessment of Functioning (GAF (Raballo et al., 2016; Comparelli et al., 2016)), evidence linking ASEs to real-world community functioning or performance-based capacity variables remains limited. In our study, only the IPASE Cognition subscale was associated with PSP in the ASD group, but this effect did not survive FDR correction. These findings indicate that while self-disturbances may not broadly relate to functional impairment, specific experiential domains (e.g., cognitive anomalies) could show context-dependent associations with real-world functioning.

### 4.5 Limitations, strengths and future directions

Several limitations should be acknowledged. The cross-sectional design precludes inference about the directionality of ASE-variable relationships. The demographic homogeneity of the sample and exclusion of individuals with intellectual disability constrain generalizability, and modest sample sizes may have reduced power to detect group differences and interaction effects. Although no participant in the ASD group met MINI criteria for a current or past psychotic disorder, the ASD group was not assessed with PSYCHS for CHR-P criteria. Therefore, subthreshold psychosis-risk symptoms cannot be fully ruled out and may have contributed to overlap in IPASE scores. Additionally, the use of prescribed stimulants, which was more common in the ASD group, may have influenced results. However, sensitivity analyses showed no change in findings, and retaining these participants may enhance ecological validity given the high co-occurrence of ADHD and ASD (Eaton et al., 2023). The majority of empirical evidence on ASEs in psychosis-spectrum populations stems from studies using the EASE. As the IPASE is a self-report measure developed independently, differences in construct operationalization and depth may influence the strength and specificity of associations observed. Also, self-report measures are susceptible to interpretation bias, including literal or rule-based response styles, which differentially affect reporting across groups (Kalandadze et al., 2018). Measurement of functioning also had limitations, as PSP ratings were based on participant interview only, without collateral informant information, which may have limited contextual information about everyday functioning. Lastly, emotion processing, a key social cognitive domain (Pinkham et al., 2014), was not assessed.

Despite these limitations, this study benefits from methodologically consistent assessments across groups, antipsychotic-free CHR-P participants, and a CC sample reflecting normative variation. Balanced sex representation also enhances generalizability.

Future longitudinal studies should investigate how ASEs, social cognition, and social functioning covary over time and whether ASEs are antecedent to, or concurrent with, changes in social cognition. Integration of clinician-rated phenomenological assessments with self-report measures could strengthen the precision and interpretability of ASE assessments across clinical populations. Further refinement of the IPASE for use across clinical groups, including normative cutoffs, may enhance clinical screening.

In conclusion, our study found ASE levels followed a graded pattern, highest in CHR—P, intermediate in ASD, and lowest in controls, consistent with phenomenological models of self-disturbances. ASEs were most consistently associated with social cognitive deficits in the ASD group, rather than in CHR—P. These findings indicate that similar symptom expressions may be observed across clinical groups, yet their cognitive and experiential correlates may differ, underscoring the importance of contextualized interpretation in transdiagnostic research.

## CRediT authorship contribution statement

**Kristina Ballestad Gundersen:** Writing – original draft, Visualization, Project administration, Methodology, Investigation, Formal analysis, Data curation, Conceptualization. **Tina Dam Kristensen:** Writing – review & editing, Supervision, Methodology. **Alberte C.E. Jeppesen:** Writing – review & editing, Investigation. **Johannes Andresen:** Writing – review & editing, Investigation. **Helle Gade Andersen:** Writing – review & editing, Investigation. **Birgitte Fagerlund:** Writing – review & editing. **Paolo Fusar-Poli:** Writing – review & editing. **Merete Nordentoft:** Writing – review & editing, Funding acquisition. **Bjørn H. Ebdrup:** Writing – review & editing, Funding acquisition. **Scott W. Woods:** Funding acquisition. **Martha E. Shenton:** Data curation, Funding acquisition. **Barnaby Nelson:** Funding acquisition, Writing – review & editing. **Louise Birkedal Glenthøj:** Conceptualization, Funding acquisition, Methodology, Supervision, Writing – review & editing.

## Declaration of Generative AI and AI-assisted technologies in the writing process

During the preparation of this work the authors used ChatGPT in order to improve readability of the article. After using this tool, the authors reviewed and edited the content as needed and takes full responsibility for the content of the published article.

## Funding

This study was supported by grants from two independent projects. The Accelerating Medicines Partnership® Schizophrenia (AMP® SCZ) is a public-private partnership managed by the Foundation for the National Institutes of Health. The AMP SCZ research program is supported by contributions from the AMP SCZ public and private partners, which include the 10.13039/100000025National Institute of Mental Health (NIMH: U24MH124629, U01MH124631, and U01MH124639) and 10.13039/100010269Wellcome (220664/Z/20/Z and 220664/A/20/Z). The STEPS trial granted funding from the 10.13039/501100003554Lundbeck Foundation (grant number: R396-2022-88) and The 10.13039/501100004836Independent Research Fund Denmark (grant number: 2096-00078B).

## Declaration of competing interest

LBG has received lecture fees from Boehringer Ingelheim, Lundbeck Pharma, and Heka-VR. BE is part of the Advisory Board of Boehringer Ingelheim, Lundbeck Pharma A/S; and has received lecture fees from Boehringer Ingelheim, Lundbeck Pharma A/S, Otsuka Pharma Scandinavia AB, and Teva Pharmaceuticals. HGA has received lecture fees from Lundbeck Pharma. All other authors have no declaration of interests.

## Data Availability

Curated AMP® SCZ data are available through the NIMH Data Archive (NDA) to qualified researchers under an approved Data Use Certification, subject to NDA access requirements. Data from the site-specific add-on protocol are available upon reasonable request.

## References

[bb0005] Addington J. (2008). Social functioning in individuals at clinical high risk for psychosis. Schizophr. Res..

[bb0010] Allswede D.M. (2020). Characterizing covariant trajectories of individuals at clinical high risk for psychosis across symptomatic and functional domains. Am. J. Psychiatry.

[bb0015] An S.K. (2010). Attribution bias in ultra-high risk for psychosis and first-episode schizophrenia. Schizophr. Res..

[bb0020] Andresen J. (2025). The effect of virtual reality-based social cognitive training for autistic adults: a study protocol for STEPS (social cognitive training enhancing pro-functional skills), a randomised clinical trial. JMIR. Res. Protoc..

[bb0025] Axelrod B.N. (2002). Validity of the Wechsler abbreviated scale of intelligence and other very short forms of estimating intellectual functioning. Assessment.

[bb0030] Barbato M. (2015). Theory of mind, emotion recognition and social perception in individuals at clinical high risk for psychosis: findings from the NAPLS-2 cohort. Schizophr. Res. Cogn..

[bb0035] Baron-Cohen S. (2005). The Adult Asperger Assessment (AAA): a diagnostic method. J. Autism Dev. Disord..

[bb0040] Bishop-Fitzpatrick L. (2015). The relationship between stress and social functioning in adults with autism spectrum disorder and without intellectual disability. Autism Res..

[bb0045] Bruni T.P. (2014). Test review: Social Responsiveness Scale–Second Edition (SRS-2). J. Psychoeduc. Assess..

[bb0050] Cicero D.C. (2016). Anomalous self-experiences and positive symptoms are independently associated with emotion processing deficits in schizophrenia. Schizophr. Res..

[bb0055] Cicero D.C. (2017). The Inventory of Psychotic-Like Anomalous Self-Experiences (IPASE): development and validation. Psychol. Assess..

[bb0060] Cobanovic H. (2025). Validity of self-rating questionnaires used for assessing self-disorders? A systematic review. Psychopathology.

[bb0065] Comparelli A. (2016). Anomalous self-experiences and their relationship with symptoms, neuro-cognition, and functioning in at-risk adolescents and young adults. Compr. Psychiatry.

[bb0070] Devoe D.J. (2020). Negative symptoms and functioning in youth at risk of psychosis: a systematic review and meta-analysis. Harv. Rev. Psychiatry.

[bb0075] Didehbani N. (2012). Brief report: insight into illness and social attributional style in Asperger’s syndrome. J. Autism Dev. Disord..

[bb0080] Eaton C. (2023). The prevalence of attention deficit/hyperactivity disorder symptoms in children and adolescents with autism spectrum disorder without intellectual disability: a systematic review. J. Atten. Disord..

[bb0085] Ebisch S.J.H. (2013). Out of touch with reality? Social perception in first-episode schizophrenia. Soc. Cogn. Affect. Neurosci..

[bb0090] First M. (2016). Structured clinical interview for DSM-5 personality disorders (SCID-5-PD). Testkatalog.

[bb0095] van der Gaag M. (2013). Development of the Davos Assessment of Cognitive Biases Scale (DACOBS). Schizophr. Res..

[bb0100] Glenthøj L.B. (2016). Social cognition in patients at ultra-high risk for psychosis: what is the relation to social skills and functioning?. Schizophr. Res. Cogn..

[bb0105] Green M.F. (2000). Neurocognitive deficits and functional outcome in schizophrenia: are we measuring the “right stuff”?. Schizophr. Bull..

[bb0110] Haug E. (2014). Anomalous self-experiences contribute independently to social dysfunction in the early phases of schizophrenia and psychotic bipolar disorder. Compr. Psychiatry.

[bb0115] Henriksen M.G., Raballo A., Nordgaard J. (2021). Self-disorders and psychopathology: a systematic review. Lancet Psychiatry.

[bb0120] Honan C.A. (2016). The awareness of social inference test: development of a shortened version for use in adults with acquired brain injury. Clin. Neuropsychol..

[bb0125] Jeličić A. (2025). Autism and schizophrenia spectrum disorder: phenomenological qualitative study of patients' experience. Front. Psychol..

[bb0130] Jeppesen A.C.E. (2025). Study protocol for the EYEdentify project: an examination of gaze behaviour in autistic adults using a virtual reality-based paradigm. PLoS One.

[bb0135] Kalandadze T. (2018). Figurative language comprehension in individuals with autism spectrum disorder: a meta-analytic review. Autism.

[bb0140] Kinnaird E., Stewart C., Tchanturia K. (2019). Investigating alexithymia in autism: a systematic review and meta-analysis. Eur. Psychiatry.

[bb0145] Larson F.V. (2017). Psychosis in autism: comparison of the features of both conditions in a dually affected cohort. Br. J. Psychiatry.

[bb0150] Lord C. (2012).

[bb0155] Magnani F. (2023). The Inventory of Psychotic-Like Anomalous Self-Experiences (IPASE): an easy tool for investigating self-disorders, subjective experiences and global functioning. Eur. Psychiatry.

[bb0160] McDonald S. (2006). Reliability and validity of The Awareness of Social Inference Test (TASIT): a clinical test of social perception. Disabil. Rehabil..

[bb0165] Morel-Kohlmeyer S. (2021). When alterations in social cognition meet subjective complaints in autism spectrum disorder: evaluation with the "ClaCoS" battery. Front. Psychol..

[bb0170] Nelson B. (2009). Does disturbance of self underlie social cognition deficits in schizophrenia and other psychotic disorders?. Early Interv. Psychiatry.

[bb0175] Nelson B. (2019). The construct validity of the Inventory of Psychotic-Like Anomalous Self-Experiences (IPASE) as a measure of minimal self-disturbance: preliminary data. Early Interv. Psychiatry.

[bb0180] Nilsson M. (2019). Arguments for a phenomenologically informed clinical approach to autism spectrum disorder. Psychopathology.

[bb0185] Nilsson M. (2020). Self-disorders in Asperger syndrome compared to schizotypal disorder: a clinical study. Schizophr. Bull..

[bb0190] Oliver L.D. (2021). Social cognitive performance in schizophrenia spectrum disorders compared with autism spectrum disorder: a systematic review, meta-analysis, and meta-regression. JAMA Psychiatr..

[bb0195] Orsmond G.I. (2013). Social participation among young adults with an autism spectrum disorder. J. Autism Dev. Disord..

[bb0200] Ozbek S.U., Sut E., Bora E. (2023). Comparison of social cognition and neurocognition in schizophrenia and autism spectrum disorder: a systematic review and meta-analysis. Neurosci. Biobehav. Rev..

[bb0205] Parnas J., Henriksen M.G. (2014). Disordered self in the schizophrenia spectrum: a clinical and research perspective. Harv. Rev. Psychiatry.

[bb0210] Parnas J. (2005). EASE: examination of anomalous self-experience. Psychopathology.

[bb0215] Patterson T.L. (2001). Social skills performance assessment among older patients with schizophrenia. Schizophr. Res..

[bb0220] Pinkham A.E. (2014). The social cognition psychometric evaluation study: results of the expert survey and RAND panel. Schizophr. Bull..

[bb0225] Pinkham A.E. (2020). Comprehensive comparison of social cognitive performance in autism spectrum disorder and schizophrenia. Psychol. Med..

[bb0230] Piskulic D. (2016). Social cognition over time in individuals at clinical high risk for psychosis: findings from the NAPLS-2 cohort. Schizophr. Res..

[bb0235] Raballo A. (2016). Self-disorders and clinical high risk for psychosis: an empirical study in help-seeking youth attending community mental health facilities. Schizophr. Bull..

[bb0240] Raballo A. (2021). The self in the spectrum: a meta-analysis of the evidence linking basic self-disorders and schizophrenia. Schizophr. Bull..

[bb0245] Rashidi A.G. (2025). Comparative analysis of social cognitive and neurocognitive performance across autism and schizophrenia spectrum disorders. Schizophr. Bull..

[bb0250] Recio P. (2024). Autistic sensory traits and psychological distress: mediating role of worry and intolerance of uncertainty. Brain Sci..

[bb0255] Rutter M., Le Couteur A., Lord C. (2003).

[bb0260] Sass L.A., Parnas J. (2003). Schizophrenia, consciousness, and the self. Schizophr. Bull..

[bb0265] Sasson N.J. (2011). The benefit of directly comparing autism and schizophrenia for revealing mechanisms of social cognitive impairment. J. Neurodev. Disord..

[bb0270] Sasson N.J. (2020). Social cognition as a predictor of functional and social skills in autistic adults without intellectual disability. Autism Res..

[bb0275] Scheeren A.M. (2022). Objective and subjective psychosocial outcomes in adults with autism spectrum disorder: a 6-year longitudinal study. Autism.

[bb0280] Sheehan D.V. (1998). The mini-international neuropsychiatric interview (M.I.N.I.): the development and validation of a structured diagnostic psychiatric interview for DSM-IV and ICD-10. J. Clin. Psychiatry.

[bb0285] Spain D., Sin J., Freeman D. (2016). Conceptualising paranoia in ASD: a systematic review and development of a theoretical framework. Res. Autism Spectr. Disord..

[bb0290] Værnes T.G., Røssberg J.I., Møller P. (2019). Anomalous self-experiences are strongly associated with negative symptoms in a clinical high-risk for psychosis sample. Compr. Psychiatry.

[bb0295] Vaquerizo-Serrano J. (2022). Autism spectrum disorder and clinical high risk for psychosis: a systematic review and meta-analysis. J. Autism Dev. Disord..

[bb0300] Velikonja T., Fett A.-K., Velthorst E. (2019). Patterns of nonsocial and social cognitive functioning in adults with autism spectrum disorder: a systematic review and meta-analysis. JAMA Psychiatr..

[bb0305] Wannan C.M.J. (2024). Accelerating Medicines Partnership® Schizophrenia (AMP® SCZ): rationale and study design of the largest global prospective cohort study of clinical high risk for psychosis. Schizophr. Bull..

[bb0310] White S. (2016). The reliability of the Personal and Social Performance scale - informing its training and use. Psychiatry Res..

[bb0315] Wilson C.S. (2020). Feasibility of psychosis risk assessment for adolescents diagnosed with autism. Autism.

[bb0320] Woods S.W. (2024). Development of the PSYCHS: positive SYmptoms and diagnostic criteria for the CAARMS harmonized with the SIPS. Early Interv. Psychiatry.

