# Supplementary material for: Comparative profiles of anomalous self-experiences and social cognition in clinical high risk for psychosis and autism spectrum disorder
Source: Schizophr Res Cogn. 2026 Jun 11;46:100450. doi: 10.1016/j.scog.2026.100450 (PMC13272541; doi:10.1016/j.scog.2026.100450)
Supplement: Supplementary file 1 — Supplementary material [file mmc1.docx]

**Appendices**

**Appendix A**. Supplementary Methods

**Table A.1** *Eligibility criteria for all participants*

|  | **AMP® SCZ / PRESCIENT** | | **STEPS/EYEdentify** | |
| --- | --- | --- | --- | --- |
| **Domain** | **CHR-P** | **Controls (community controls)** | **ASD** | **Controls (healthy controls)** |
| Recruitment source | Psychiatric services (Copenhagen area) | General population | Psychiatric services (Copenhagen area) | General population |
| Harmonized age in present study | 18–30 years | 18–30 years | 18–30 years | 18–30 years |
| Group definition (inclusion) | Meets CHR-P criteria operationalized via PSYCHS (CAARMS/SIPS harmonized) | Does not meet CHR-P criteria | Documentation of pre-existing ICD-10 ASD diagnosis (F84.0, F84.1, F84.5, F84.8) and current social impairment (SRS-2, T-score ≥60) | Neurotypical controls with no current psychiatric disorder |
| Cluster A personality disorders (Paranoid/Schizoid/Schizotypal) | Assessed; not an exclusion criterion for CHR-P | **Excluded** if current or past Cluster A PD | Not assessed for eligibility in this cohort | Not assessed for eligibility in this cohort |
| Family history of psychotic spectrum disorders (first-degree) | Assessed; not an exclusion criterion for CHR-P | Assessed; **excluded** if positive first-degree family history | Not assessed for eligibility in this cohort | Not assessed for eligibility in this cohort |
| Psychotic disorder | **Excluded** if current or past psychotic disorder via PSYCHS criterion | **Excluded** if current or past psychotic disorder via PSYCHS criterion | Not an exclusion criterion. MINI used to characterize comorbidity. In this sample, no participants met MINI criteria for current or past psychotic disorder | **Excluded** via MINI criterion for any current or past psychiatric disorder |
| Other current psychiatric disorders other than those listed above | Allowed, except exclusions below (e.g., psychotic disorder) | Allowed (community controls), except exclusions below | Allowed, except STEPS protocol exclusions below | **Excluded** if any current MINI psychiatric diagnosis (including alcohol/drug dependence) |
| Antipsychotic exposure | **Excluded** if current or lifetime exposure equivalent to a total lifetime haloperidol dose of >50 mg | **Excluded** if current or lifetime exposure equivalent to a total lifetime haloperidol dose of >50 mg | Allowed. Not a STEPS inclusion criterion | Not an explicit exclusion; indirectly constrained by “no current psychiatric disorder” |
| Other psychotropic medication usage | Allowed | **Excluded** if any current psychotropic medication | Allowed. ADHD medication requirements apply (below) | Not an explicit exclusion; indirectly constrained by “no current psychiatric disorder” |
| Intellectual disability / IQ | **Excluded** if documented history of intellectual disability (Estimated IQ < 70) | **Excluded** if documented history of intellectual disability (Estimated IQ < 70) | **Excluded** if documented history of intellectual disability (Estimated IQ < 70) | **Excluded** if documented history of intellectual disability (Estimated IQ < 70) |
| Current social impairment | Not assessed for eligibility in this cohort | Not assessed for eligibility in this cohort | **Excluded** if T-score of ≥60 on the Social Responsiveness Scale Total Score, Second Edition, Adult Form | Not assessed for eligibility in this cohort |
| Organic brain disease / central nervous system disorder | **Excluded** if past or current clinically relevant central nervous system disorder | **Excluded** if past or current clinically relevant central nervous system disorder | **Excluded** for organic brain disease (ICD-10 F00-F09) | **Excluded** for organic brain disease (ICD-10 F00-F09) |
| Traumatic brain injury (TBI) | **Excluded** if TBI screening instrument score ≥7 | **Excluded** if TBI screening instrument score ≥7 | Allowed; Not assessed for eligibility in this cohort | Allowed; Not assessed for eligibility in this cohort |
| Language proficiency (Danish/English) | **Excluded** if inadequate Danish/English | **Excluded** if inadequate Danish/English | **Excluded** if inadequate Danish/English for assessments/intervention | **Excluded** if inadequate Danish/English |
| ADHD-related criteria | Not assessed for eligibility in this cohort | Not assessed for eligibility in this cohort | If ADHD present: must have satisfactory treatment response; no medication changes within 4 weeks; excluded if medically untreated symptoms | Excluded if ADHD meets criteria as a current MINI psychiatric diagnosis |
| Immediate risk of homicide/suicide | Not assessed for eligibility in this cohort | Not assessed for eligibility in this cohort | **Excluded** if immediate risk of homicide or suicide | Not an explicit exclusion; indirectly constrained by “no current psychiatric disorder” |
| Control definition summary | — | **Community controls:** psychiatric diagnoses may be present, but excluded for CHR status, current/past psychotic disorder, current/past Cluster A PD, current psychotropic medication, and first-degree psychosis family history | — | **Healthy controls:** excluded for any current MINI psychiatric disorder and insufficient Danish/English |

***Note.*** Study criteria are summarized from the AMP® SCZ/PRESCIENT and STEPS/EYEdentify protocols (Andresen et al., 2025; Jeppesen et al., 2025; Wannan et al., 2024). Harmonized restrictions applied in the present study include age 18–30 years, estimated IQ >70, and no self-reported organic brain disease (ICD-10 F00–F09).

**Table A.2** *Screening and diagnostic instruments administered by cohort and group*

|  |  | **AMP® SCZ / PRESCIENT** | | **STEPS/EYEdentify** | |
| --- | --- | --- | --- | --- | --- |
| **Instrument / ascertainment method** | **Administration / modality** | **CHR-P** | **Controls (community controls)** | **ASD** | **Controls (healthy controls)** |
| PSYCHS (CHR-P ascertainment) | Clinician administered structured interview | Yes | Yes | No | No |
| SCID-5-PD (Cluster A PD assessment) | Clinician administered structured diagnostic interview | Yes | Yes | No | No |
| FIGS (family history of depression, mania, and psychosis spectrum disorders) | Clinician administered interview | Yes | Yes | No | No |
| SOFAS (current and past social and occupational functioning) | Clinician administered interview | Yes | Yes | No | No |
| MINI (current psychiatric diagnoses) | Clinician administered structured diagnostic interview | No | No | Yes | Yes |
| ICD-10 ASD diagnosis confirmation | Clinical record review (hospital medical files) | No | No | Yes | No |
| SRS-2 Adult Self-report (eligibility threshold in STEPS) | Self-report questionnaire | No | No | Yes | No |
| WAIS-IV (Vocabulary + Matrix Reasoning) for estimated IQ | Clinician administered cognitive testing (standardized subtests) | Yes | Yes | Yes | Yes |
| TBI screening instrument | Clinician administered structured screening interview / questionnaire | Yes | Yes | No | No |
| Central nervous system disorder, present study restriction | Participant self-report | Yes | Yes | No | No |
| Organic brain disease (ICD-10 F00–F09), present study restriction | Participant self-report | No | No | Yes | Yes |

***Note.*** FIGS, Family Interview for Genetic Studies; MINI, Mini-International Neuropsychiatric Interview; PSYCHS, Positive SYmptoms and Diagnostic Criteria for the CAARMS Harmonized with the SIPS; SCID-5-PD, Structured Clinical Interview for DSM-5 Personality Disorders; SOFAS, Social and Occupational Functioning Assessment Scale; SRS-2, Social Responsiveness Scale-Second Edition; TBI, traumatic brain injury; WAIS-IV, Wechsler Adult Intelligence Scale-Fourth Edition; CHR-P, clinical high risk for psychosis; ASD, autism spectrum disorder.

**Appendix B.** Robustness and Sensitivity Analyses

**Table B.1** *Sensitivity analysis comparing unadjusted ANOVA and ANCOVA adjusting for sex, age and IQ on all variables of interest*

| **Variable** | **Unadjusted 95% CI, p-value** | **Adjusted 95% CI, p- value** |
| --- | --- | --- |
| IPASE | *p* < **0.001***, F = 44.837 | *p* < **0.001***, F = 42.515 |
| DACOBS | *p* < **0.001***, F = 37.660 | *p* < **0.001***, F = 34.541 |
| TASIT-S | *p =* 0.510, F = 0.678 | *p =* 0.453, F = 0.799 |
| SSPA | *p <* **0.001***, F = 31.256 | *p <* **0.001***, F = 27.849 |
| PSP | *p <* **0.001***, F = 76.333 | *p <* **0.001***, F = 62.042 |

***Note.*** This sensitivity analysis assess the potential impact of adjusting for covariates, including missing IQ data (n = 7). Results remained consistent with the main analysis (which adjusted for age, sex, and IQ), with no meaningful changes in statistical significance, effect sizes, or interpretation across all variables.

IPASE, the inventory for psychotic like anomalous self experiences; DACOBS, davos assessment of cognitive biases scale; TASIT-S, the awareness of social inference test - short form; PSP, personal and social performance scale; SSPA, social skills performance assessment.

* Represents significance after Benjamini-Hochberg false discovery rate correction for multiple testing**.**

**Table B.2** *Sensitivity analysis on group differences in IPASE and social cognition scores (ANCOVA adjusted for age and sex assigned at birth).*

|  |  |  | **Post-hoc FDR corrected, ΔM, 95% CI, *p*** | | |
| --- | --- | --- | --- | --- | --- |
| **Variables** | **F (df_1_, df_2_), *p*** | **Partial η²** | **CHR-P vs ASD** | **CHR-P vs CC** | **ASD vs CC** |
| IPASE |  |  |  |  |  |
| Total score | F (2, 103) = 45.684, ***p* < 0.001*** | .470 | ΔM = 42.515; 26.720, 58.309; ***p* < 0.001*** | ΔM = 80.681; 63.843, 97.519; ***p*** < 0**.001*** | ΔM = 38.167; 21.279, 55.054; ***p* < 0.001*** |
| Cognition | F (2, 103) = 16.254, ***p* < 0.001*** | .240 | ΔM = 3.446; 1.538, 5.355; ***p* < 0.001*** | ΔM = 5.738; 3.704, 7.773; ***p* < 0.001*** | ΔM = 2.292; 0.251, 4.332; ***p* = 0.028*** |
| Self-awareness and presence | F (2, 103) = 48.080, ***p* < 0.001*** | .483 | ΔM = 20.679; 13.608, 27.749; ***p* < 0.001*** | ΔM = 36.860; 29.322, 44.398; ***p* < 0.001*** | ΔM = 16.181; 8.621, 23.741; ***p* < 0.001*** |
| Consciousness | F (2, 103) = 26.919, ***p* < 0.001*** | .342 | ΔM = 4.335; 1.798, 6.871; ***p* < 0.001*** | ΔM = 10.004; 7.300, 12.707; ***p* < 0.001*** | ΔM = 5.669; 2.957, 8.381; ***p* < 0.001*** |
| Somatization | F (2, 103) = 33.055, ***p* < 0.001*** | .391 | ΔM = 10.298; 5.375, 15.221; ***p* < 0.001*** | ΔM = 21.488; 16.240, 26.736; ***p* < 0.001*** | ΔM = 11.190; 5.926, 16.453; ***p* < 0.001*** |
| Demarcation/ Transitivism | F (2, 103) = 35.002, ***p* < 0.001*** | .405 | ΔM = 3.757; 2.272, 5.241; ***p* < 0.001*** | ΔM = 6.591; 5.009, 8.174; ***p* < 0.001*** | ΔM = 2.835; 1.248, 4.422; ***p* < 0.001*** |
| DACOBS | F (2, 102) = 37.276, ***p* < 0.001*** | .422 | ΔM = 3.189; -7.722, 14.099; *p* = 0.563 | ΔM = 46.075; 34.514, 57.635; ***p* < 0.001*** | ΔM = 42.886; 31.226, 54.546; ***p* < 0.001*** |
| TASIT-S | F (2, 103) = 0.722, *p =* .453 | .014 | ΔM = -0.434; -1.958, 1.090; *p* = 0.573 | ΔM = -0.984; - 2.609, 0.640; *p* = 0.232 | ΔM = - 0.550; - 2.179, 1.079; *p* = 0.505 |
| SSPA | F (2, 102) = 30.163, ***p* < 0.001*** | .372 | ΔM = 6.766; 3.226, 10.306; ***p* < 0.001*** | ΔM = -8.048; -11.799, -4.297; ***p* < 0.001*** | ΔM = -14.814; -18.597, -11.030; ***p* < 0.001*** |
| PSP | F (2, 103) = 76.034, ***p* < 0.001*** | .596 | ΔM = -0.550; -6.472, 5.373; *p* = 0.854 | ΔM = -35.009; -41.323, -28.695; ***p* < 0.001*** | ΔM = -34.459; -40.792, -28.127; ***p* < 0.001*** |

***Note.*** This sensitivity analysis excluded IQ as a covariate to assess the potential impact of missing IQ data (n = 7). Results remained consistent with the main analysis (which adjusted for age, sex, and IQ), with no meaningful changes in statistical significance, effect sizes, or interpretation across all variables.

CHR-P, clinical high risk for psychosis; ASD, autism spectrum disorder; CC, community controls; IPASE, the inventory for psychotic like anomalous self experiences; DACOBS, davos assessment of cognitive biases scale; TASIT-S, the awareness of social inference test - short form; PSP, personal and social performance scale; SSPA, social skills performance assessment.

* Represents significance after Benjamini-Hochberg false discovery rate correction for multiple testing**.**

**Table B.3** *Sensitivity analysis on group differences in IPASE and social cognition scores (ANCOVA adjusted for age, sex assigned at birth, estimated IQ and stimulant use).*

|  |  |  | **Post-hoc FDR corrected, ΔM, 95% CI, *p*** | | |
| --- | --- | --- | --- | --- | --- |
| **Variables** | **F (df_1_, df_2_), *p*** | **Partial η²** | **CHR-P vs ASD** | **CHR-P vs CC** | **ASD vs CC** |
| IPASE | F (2, 94) = 44.430, ***p* < 0.001*** | .486 | ΔM = 36.742; 17.612, 55.872; ***p* < 0.001*** | ΔM = 86.043; 67.883, 104.202; ***p* < 0.001*** | ΔM = 49.301; 27.964, 70.638; ***p* < 0.001*** |
| DACOBS | F (2, 93) = 39.163, ***p* < 0.001*** | .457 | ΔM = -5.734; -18.776, 7.308; *p* = 0.385 | ΔM = 49.381; 37.101, 61.660; ***p* < 0.001*** | ΔM = 55.115; 40.555, 69.675; ***p* < 0.001*** |
| TASIT-S | F (2, 94) = 0.722, *p =* .453 | .013 | ΔM = -0.484; -2.317, 1.348; *p* = 0.601 | ΔM = -0.964; - 2.703, 0.776; *p* = 0.274 | ΔM = - 0.479; -2.523, 1.565; *p* = 0.643 |
| SSPA | F (2, 93) = 27.080, ***p* < 0.001*** | .368 | ΔM = 9.405; 5.137, 13.673; ***p* < 0.001*** | ΔM = -8.251; -12.270, -4.233; ***p* < 0.001*** | ΔM = -17.656; -22.421, -12.891; ***p* < 0.001*** |
| PSP | F (2, 94) = 61.248, ***p* < 0.001*** | .566 | ΔM = 0.958; -6.263, 8.179; *p* = 0.793 | ΔM = -35.525; -42.379, -28.670; ***p* < 0.001*** | ΔM = -36.482; -44.537, -28.428; ***p* < 0.001*** |

***Note.*** This sensitivity analysis included stimulant use as a covariate to assess the potential impact of differences in stimulant use across groups. Results remained consistent with the main analysis (which adjusted for age, sex, and IQ), with no meaningful changes in statistical significance, effect sizes, or interpretation across all variables.

CHR-P, clinical high risk for psychosis; ASD, autism spectrum disorder; CC, community controls; IPASE, the inventory for psychotic like anomalous self experiences; DACOBS, davos assessment of cognitive biases scale; TASIT-S, the awareness of social inference test - short form; PSP, personal and social performance scale; SSPA, social skills performance assessment.

* Represents significance after Benjamini-Hochberg false discovery rate correction for multiple testing**.**

**Table B.4** *Case-handling and robustness summary in bootstrapped hierarchical linear regression models (complete-case analysis)*

| **Variable** | **N (full analysis sample)** | **Cook’s D flag threshold ^a^** | **Excluded in sensitivity model (flagged Cook’s D)** | **N (sensitivity sample)** | **Primary estimate reported in main table (Full vs Sensitivity)** | **Did key conclusions change in sensitivity?** | **Comments** |
| --- | --- | --- | --- | --- | --- | --- | --- |
| DACOBS | 100 | 0.0439 | 4 | 96 | Full | No | Results were robust to exclusion of influential cases. However, interaction parameters closed to significance. |
| TASIT-S | 101 | 0.0435 | 9 | 92 | Full | No | Results were robust to exclusion of influential cases. |
| PSP | 101 | 0.0435 | 5 | 96 | Full | Yes | Influence-sensitive; exclusion of influential cases changes conclusions, but these did not survive FDR correction. |
| SSPA | 100 | 0.0439 | 5 | 95 | Full | No | Results were robust to exclusion of influential cases. |

***Note.*** The table show, for each variable, (a) analysis N, (b) how many cases were flagged/excluded, (c) which version was reported in the main table, and (d) whether conclusions changed.

DACOBS, davos assessment of cognitive biases scale; TASIT-S, the awareness of social inference test - short form; PSP, personal and social performance scale; SSPA, social skills performance assessment.

^a^ Cook`s D threshold based on 4/(n−k−1). Number of k (predictors in final model) was 8 in all analyses.

**Table B.5** *Bootstrapped hierarchical linear regression models: comparison of full model vs. sensitivity analysis excluding influential observations (Cook’s Distance)*

|  |  |  | **Conditional slope of IPASE,**  **B [95% CI], *p*** | | | **ΔSlope vs CHR-P,**  **B [95% CI], *p*** | |  |  |
| --- | --- | --- | --- | --- | --- | --- | --- | --- | --- |
| **Variable** | **Model version** | **N** | **CHR-P** | **ASD** | **CC** | **ASD vs CHR-P** | **CC vs CHR-P** | **R² (full model)** | **ΔR² (interaction)** |
| DACOBS | Full model | 100 | 0.199 (-0.030, 0.413), p = 0.091 | 0.392 (0.236, 0.544), p < **0.001*** | 0.324 (-0.217, 0.756), p = 0.122 | 0.193 (-0.084, 0.478), p = 0.162 | 0.125 (-0.441, 0.649), p = 0.598 | .562 | .010 |
| DACOBS | Sensitivity (excl. flagged) | 96 | 0.150 (-0.069, 0.383), p = 0.191 | 0.419 (0.278, 0.570), p < **0.001*** | 0.542 (0.126, 0.916), p < **0.001*** | 0.269 (-0.009, 0.540), p = 0.052 | 0.392 (-0.070, 0.828), p = 0.057 | .608 | .023 |
| TASIT-S | Full model | 101 | 0.002 (-0.038, 0.037), p = 0.914 | 0.001 (-0.026, 0.032), p = 0.932 | -0.013 (-0.135, 0.055), p = 0.743 | 0.000 (-0.048, 0.050), p = 0.985 | -0.015 (-0.130, 0.061), p = 0.739 | .055 | .001 |
| TASIT-S | Sensitivity (excl. flagged) | 92 | 0.014 (-0.015, 0.037), p = 0.328 | 0.016 (-0.008, 0.041), p = 0.164 | -0.014 (-0.105, 0.042), p = 0.639 | 0.002 (-0.036, 0.045), p = 0.906 | -0.028 (-0.125, 0.041), p = 0.398 | .175 | .007 |
| PSP | Full model | 101 | -0.019 (-0.136, 0.088), p = 0.740 | -0.143 (-0.261, -0.012), p = 0.029 | -0.096 (-0.232, 0.026), p = 0.108 | -0.123 (-0.287, 0.064), p = 0.160 | -0.077 (-0.261, 0.094), p = 0.366 | .627 | .010 |
| PSP | Sensitivity (excl. flagged) | 96 | -0.026 (-0.139, 0.074), p = 0.638 | -0.182 (-0.284, -0.079), p = 0.050 | -0.072 (-0.224, 0.036), p = 0.229 | -0.156 (-0.308, 0.007), p = 0.044 | -0.046 (-0.241, 0.120), p = 0.588 | .735 | .017 |
| SSPA | Full model | 100 | 0.022 (-0.065, 0.119), p = 0.618 | 0.006 (-0.051, 0.059), p = 0.823 | -0.010 (-0.107, 0.092), p = 0.796 | -0.016 (-0.118, 0.080), p = 0.767 | -0.032 (-0.161, 0.098), p = 0.577 | .393 | .001 |
| SSPA | Sensitivity (excl. flagged) | 95 | 0.016 (-0.041, 0.087), p = 0.618 | 0.014 (-0.040, 0.067), p = 0.574 | -0.002 (-0.096, 0.091), p = 0.945 | -0.002 (-0.089, 0.073), p = 0.964 | -0.018 (-0.130, 0.080), p = 0.706 | .502 | .000 |

***Note***. This table presents full-sample estimates (primary) and sensitivity estimates excluding observations flagged by Cook’s distance in the full-sample final model. Sensitivity analyses were conducted once per variable; no iterative case-removal procedure was applied. Bootstrapped hierarchical linear regression models were estimated separately for each variable. Covariates (age, sex, and estimated IQ) were entered in Block 1; grand-mean centered IPASE and group (dummy coded with CHR-P as the reference category) were entered in Block 2; and IPASE×group interaction terms were entered in Block 3. Unstandardized coefficients (B) with bias-corrected and accelerated (BCa) bootstrapped 95% confidence intervals based on 5000 stratified resamples by group (fixed random seed) are reported; p-values are two-sided. Group-specific IPASE slopes (CHR-P, ASD, CC) are conditional effects derived from the Block 3 interaction model (with slopes for ASD and CC obtained by re-estimating the model using alternative reference groups). Model fit is summarized using R² for the full model (Block 3) and ΔR² for the interaction block (Block 3 minus Block 2). Regression assumptions were evaluated for each final model using normality plots (Q–Q/P–P plots and histograms), residual-versus-fitted plots to assess linearity and homoscedasticity, and collinearity diagnostics (tolerance/VIF). Potential outliers and influential observations were examined using standardized residuals (|z| > 3) and Cook’s distance, applying the threshold 4/(n - k - 1).

* Represents significance after Benjamini-Hochberg false discovery rate correction for multiple testing

**Table B.6** *Bootstrapped hierarchical linear regression models: comparison of full model vs. sensitivity analysis without estimated IQ as covariate vs sensitivity analysis adding stimulant use as covariate*

|  |  |  | **Conditional slope of IPASE,**  **B [95% CI], *p*** | | | **ΔSlope vs CHR-P,**  **B [95% CI], *p*** | |  |  |
| --- | --- | --- | --- | --- | --- | --- | --- | --- | --- |
| **Variable** | **Model version** | **N** | **CHR-P** | **ASD** | **CC** | **ASD – CHR-P** | **CC – CHR-P** | **R² (full model)** | **ΔR²**  **(interaction)** |
| DACOBS | Full model | 100 | 0.199 (-0.030, 0.413), *p* = 0.091 | 0.392 (0.236, 0.544), *p* < **0.001*** | 0.324 (-0.217, 0.756*), p* = 0.122 | 0.193 (-0.084, 0.478), *p* = 0.162 | 0.125 (-0.441, 0.649), *p* = 0.598 | .562 | .010 |
| DACOBS | Sensitivity (without estimated IQ) | 107 | 0.189 (-0.029, 0.385), *p* = 0.097 | 0.389 (0.230, 0.548), *p* < **0.001*** | 0.373 (-0.090, 0.783*), p* = 0.058 | 0.200 (-0.060, 0.476), *p* = 0.134 | 0.184 (-0.338, 0.670), *p* = 0.426 | .558 | .012 |
| DACOBS | Sensitivity (including stimulant use) | 100 | 0.188 (-0.021, 0.373), *p* = 0.098 | 0.364 (0.198, 0.524), *p* < **0.001*** | 0.330 (-0.117, 0.754*), p* = 0.117 | 0.176 (-0.106, 0.481), *p* = 0.098 | 0.142 (-0.423, 0.673), *p* = 0.551 | .575 | .009 |
| TASIT-S | Full model | 101 | 0.002 (-0.038, 0.037), *p* = 0.914 | 0.001 (-0.026, 0.032), *p* = 0.932 | -0.013 (-0.135, 0.055), *p* = 0.743 | 0.000 (-0.048, 0.050), *p* = 0.985 | -0.015 (-0.130, 0.061), *p* = 0.739 | .055 | .001 |
| TASIT-S | Sensitivity (without estimated IQ) | 108 | -0.004 (-0.042, 0.028), *p* = 0.848 | 0.001 (-0.025, 0.033), *p* = 0.925 | -0.019 (-0.125, 0.046), *p* = 0.619 | 0.005 (-0.040, 0.059), *p* = 0.829 | -0.015 (-0.122, 0.063), *p* = 0.720 | .046 | .003 |
| TASIT-S | Sensitivity (including stimulant use) | 101 | 0.001 (-0.047, 0.038), *p* = 0.956 | -0.001 (-0.030, 0.029), *p* = 0.948 | -0.012 (-0.136, 0.056), *p* = 0.757 | -0.002 (-0.047, 0.046), *p* = 0.935 | -0.013 (-0.125, 0.061), *p* = 0.762 | .063 | .001 |
| PSP | Full model | 101 | -0.019 (-0.136, 0.088), *p* = 0.740 | -0.143 (-0.261, -0.012), *p* = 0.029 | -0.096 (-0.232, 0.026), *p* = 0.108 | -0.123 (-0.287, 0.064), *p* = 0.160 | -0.077 (-0.261, 0.094), *p* = 0.366 | .627 | .010 |
| PSP | Sensitivity (without estimated IQ) | 108 | -0.021 (-0.122, 0.079), *p* = 0.680 | -0.142 (-0.257, -0.013), *p* = 0.027 | -0.113 (-0.271, -0.004), *p* = 0.073 | -0.121 (-0.275, 0.045), *p* = 0.136 | -0.092 (-0.280, 0.052), *p* = 0.264 | .638 | .010 |
| PSP | Sensitivity (including stimulant use) | 101 | -0.017 (-0.133, 0.089), *p* = 0.774 | -0.136 (-0.264, -0.001), *p* = 0.051 | -0.098 (-0.231, 0.023), *p* = 0.106 | -0.120 (-0.287, 0.063), *p* = 0.186 | -0.081 (-0.268, 0.095), *p* = 0.348 | .629 | .010 |
| SSPA | Full model | 100 | 0.022 (-0.065, 0.119), *p* = 0.618 | 0.006 (-0.051, 0.059), *p* = 0.823 | -0.010 (-0.107, 0.092), *p* = 0.796 | -0.016 (-0.118, 0.080), *p* = 0.767 | -0.032 (-0.161, 0.098), *p* = 0.577 | .393 | .001 |
| SSPA | Sensitivity (without estimated IQ) | 107 | 0.021 (-0.060, 0.105), *p* = 0.606 | 0.007 (-0.054, 0.060), *p* = 0.785 | -0.019 (-0.096, 0.066), *p* = 0.588 | -0.014 (-0.109, 0.075), *p* = 0.785 | -0.040 (-0.146, 0.068), *p* = 0.459 | .382 | .001 |
| SSPA | Sensitivity (including stimulant use) | 100 | 0.026 (-0.059, 0.119), *p* = 0.546 | 0.015 (-0.045, 0.074), *p* = 0.590 | -0.012 (-0.108, 0.092), *p* = 0.755 | -0.011 (-0.111, 0.084), *p* = 0.843 | -0.040 (-0.164, 0.089), *p* = 0.506 | .406 | .001 |

***Note.*** This table display results from main analysis vs sensitivity analyses to assess the potential impact of missing IQ data (n = 7) as well impact of stimulant use. Results remained consistent with the main analysis (which adjusted for age, sex, and IQ), with no meaningful changes in statistical significance, effect sizes, or interpretation across all variables. Bootstrapped hierarchical linear regression models were estimated separately for each variable (total score) and adjusted for covariates. Values represent unstandardized regression coefficients (B) with bias-corrected and accelerated 95% confidence intervals (CI) based on 5000 stratified bootstrap resamples. Group-specific slopes (CHR-P, ASD, CC) are conditional effects from the interaction model. R² indicates full model fit. ΔR² reflects the additional variance explained by interaction terms.

CHR-P, clinical high risk for psychosis; ASD, autism spectrum disorder; CC, community controls; IPASE, the inventory for psychotic like anomalous self experiences; DACOBS, davos assessment of cognitive biases scale; TASIT-S, the awareness of social inference test - short form; PSP, personal and social performance scale; SSPA, social skills performance assessment.
* Represents significance after Benjamini-Hochberg false discovery rate correction for multiple testing.

**Table B.7** *Bootstrapped hierarchical linear regression models of IPASE subscales predicting social cognitive variables by group (complete-case analysis)*

Panel A. DACOBS total score

|  |  | **Conditional slope of IPASE,**  **B [95% CI], *p*** | | | **ΔSlope vs CHR-P,**  **B [95% CI], *p*** | |  |  |
| --- | --- | --- | --- | --- | --- | --- | --- | --- |
| **IPASE subscale predictor (centered)** | **N** | **CHR-P** | **ASD** | **CHR-P** | **ASD vs CHR-P** | **CC vs CHR-P** | **R² (full model)** | **R² change (interaction block)** |
| Cognition | 100 | -0.008 (-2.106, 1.665), *p* = 0.994 | 3.189 (1.568, 5.119), p < **0.001*** | 2.236 (-1.695, 6.214), p = 0.172 | 3.197 (0.527, 6.366), p = 0.017 | 2.244 (-1.939, 7.421), p = 0.251 | .513 | .042 |
| Self-awareness and presence | 100 | 0.318 (-0.192, 0.873), p = 0.228 | 0.780 (0.403, 1.137), p < **0.001*** | 0.893 (-0.457, 2.340), p = 0.068 | 0.462 (-0.160, 1.057), p = 0.154 | 0.575 (-0.872, 2.017), p = 0.154 | .536 | .013 |
| Consciousness | 100 | 1.373 (-0.040, 2.811), p = 0.059 | 2.544 (1.519, 3.774), p < **0.001*** | 2.076 (-0.906, 4.414), p = 0.115 | 1.171 (-0.535, 3.052), p = 0.191 | 0.703 (-2.649, 3.544), p = 0.638 | .567 | .010 |
| Somatization | 100 | 0.816 (0.071, 1.461), p = 0.030 | 1.275 (0.829, 1.752), p < **0.001*** | 0.461 (-1.004, 1.632), p = 0.488 | 0.459 (-0.406, 1.438), p = 0.291 | -0.354 (-1.953, 1.116), p = 0.291 | .564 | .010 |
| Demarcation/  Transitivism | 100 | 1.582 (-0.604, 3.436), p = 0.134 | 2.704 (0.586, 4.822), p = **0.013*** | 3.683 (-1.551, 9.137), p = 0.095 | 1.123 (-1.740, 4.298), p = 0.451 | 2.101 (-3.303, 8.069), p = 0.389 | .484 | .005 |

Panel B. PSP total score

|  |  | **Conditional slope of IPASE,**  **B [95% CI], *p*** | | | **ΔSlope vs CHR-P,**  **B [95% CI], *p*** | |  |  |
| --- | --- | --- | --- | --- | --- | --- | --- | --- |
| **IPASE subscale predictor (centered)** | **N** | **CHR-P** | **ASD** | **CHR-P** | **ASD vs CHR-P** | **CC vs CHR-P** | **R² (full model)** | **R² change (interaction block)** |
| Cognition | 101 | 0.388 (-0.402, 1.031), p = 0.285 | -1.579 (-2.824, -0.328), p = **0.017*** | -0.679 (-1.873, 0.198), p = 0.153 | -1.967 (-3.477, -0.435), p = 0.010 | -1.067 (-2.403, 0.053), p = 0.063 | .640 | .037 |
| Self-awareness and presence | 101 | -0.014 (-0.245, 0.225), p = 0.912 | -0.338 (-0.572, -0.054), p = **0.008*** | -0.247 (-0.590, 0.022), p = 0.109 | -0.324 (-0.661, 0.052), p = 0.064 | -0.233 (-0.697, 0.137), p = 0.260 | .632 | .014 |
| Consciousness | 101 | -0.536 (-1.265, 0.173), p = 0.158 | -0.757 (-1.667, 0.114), p = 0.136 | -0.838 (-1.668, 0.142), p = 0.066 | -0.221 (-1.391, 0.947), p = 0.731 | -0.302 (-1.372, 0.959), p = 0.607 | .624 | .001 |
| Somatization | 101 | -0.084 (-0.487, 0.319), p = 0.680 | -0.321 (-0.838, 0.111), p = 0.207 | -0.171 (-0.513, 0.215), p = 0.309 | -0.237 (-0.747, 0.272), p = 0.357 | -0.087 (-0.985, 0.811), p = 0.848 | .608 | .004 |
| Demarcation/  Transitivism | 101 | -0.029 (-1.049, 1.159), p = 0.959 | -1.292 (-2.710, 0.446), p = 0.113 | 0.220 (-1.354, 1.727), p = 0.722 | -1.264 (-2.990, 0.760), p = 0.201 | 0.248 (-1.494, 1.893), p = 0.751 | .609 | .011 |

Panel C. SSPA total score

|  |  | **Conditional slope of IPASE,**  **B [95% CI], *p*** | | | **ΔSlope vs CHR-P,**  **B [95% CI], *p*** | |  |  |
| --- | --- | --- | --- | --- | --- | --- | --- | --- |
| **IPASE subscale predictor (centered)** | **N** | **CHR-P** | **ASD** | **CHR-P** | **ASD vs CHR-P** | **CC vs CHR-P** | **R² (full model)** | **R² change (interaction block)** |
| Cognition | 100 | 0.481 (-0.069, 1.096), p = 0.112 | -0.082 (-0.906, 0.603), p = 0.828 | 0.078 (-0.576, 1.428), p = 0.796 | -0.563 (-1.562, 0.342), p = 0.240 | -0.404 (-1.316, 1.156), p = 0.325 | .411 | .014 |
| Self-awareness and presence | 100 | -0.070 (-0.282, 0.171), p = 0.518 | -0.010 (-0.249, 0.338), p = 0.919 | 0.049 (-0.066, 0.159), p = 0.392 | 0.119 (-0.110, 0.321), p = 0.339 | 0.060 (-0.257, 0.407), p = 0.684 | .398 | .008 |
| Consciousness | 100 | 0.257 (-0.247, 0.819), p = 0.323 | -0.152 (-0.573, 0.292), p = 0.446 | -0.041 (-0.596, 0.519), p = 0.880 | -0.409 (-1.049, 0.215), p = 0.212 | -0.298 (-1.045, 0.434), p = 0.427 | .402 | .012 |
| Somatization | 100 | 0.154 (-0.095, 0.425), P = 0.241 | -0.010 (-0.201, 0.167), p = 0.917 | -0.060 (-0.408, 0.191), p = 0.586 | -0.164 (-0.486, 0.150), P = 0.317 | -0.214 (-0.639, 0.139), P = 0.220 | .401 | .009 |
| Demarcation/  Transitivism | 100 | 0.305 (-0.421, 1.100), P = 0.424 | 0.134 (-0.656, 0.911), p = 0.732 | 0.202 (-1.731, 1.736), p = 0.798 | -0.172 (-1.236, 0.878), P = 0.757 | -0.103 (-2.102, 1.600), P = 0.914 | .396 | .001 |

Panel D. TASIT-S total score

|  |  | **Conditional slope of IPASE,**  **B [95% CI], *p*** | | | **ΔSlope vs CHR-P,**  **B [95% CI], *p*** | |  |  |
| --- | --- | --- | --- | --- | --- | --- | --- | --- |
| **IPASE subscale predictor (centered)** | **N** | **CHR-P** | **ASD** | **CHR-P** | **ASD vs CHR-P** | **CC vs CHR-P** | **R² (full model)** | **R² change (interaction block)** |
| Cognition | 101 | -0.034 (-0.315, 0.216), p = 0.809 | 0.005 (-0.282, 0.302), p = 0.974 | -0.101 (-1.124, 0.705), p = 0.763 | 0.039 (-0.350, 0.480), p = 0.855 | -0.067 (-1.045, 0.772), p = 0.853 | .055 | .001 |
| Self-awareness and presence | 101 | 0.000 (-0.090, 0.080), p = 0.993 | 0.000 (-0.061, 0.062), p = 0.995 | 0.009 (-0.327, 0.185), p = 0.926 | -0.001 (-0.103, 0.114), p = 0.991 | 0.008 (-0.313, 0.202), p = 0.939 | .053 | .000 |
| Consciousness | 101 | -0.048 (-0.302, 0.184), p = 0.680 | 0.031 (-0.164, 0.249), p = 0.774 | -0.184 (-0.815, 0.292), p = 0.518 | 0.080 (-0.231, 0.412), p = 0.629 | -0.136 (-0.818, 0.184), p = 0.668 | .064 | .010 |
| Somatization | 101 | 0.046 (-0.067, 0.141), p = 0.380 | 0.009 (-0.083, 0.101), p = 0.854 | -0.046 (-0.300, 0.107), p = 0.600 | -0.037 (-0.185, 0.123), p = 0.380 | -0.091 (-0.366, 0.106), p = 0.376 | .063 | .007 |
| Demarcation/  Transitivism | 101 | -0.077 (-0.415, 0.291), p = 0.646 | -0.031 (-0.348, 0.283), p = 0.854 | -0.193 (-0.830, 1.231), p = 0.619 | 0.046 (-0.425, 0.480), p = 0.847 | -0.116 (-0.829, 1.340), p = 0.781 | .058 | .001 |

***Note.*** Models adjusted for age, sex, and estimated IQ. Group was dummy-coded with CHR as the reference category; IPASE subscales were grand-mean centered. Unstandardized coefficients (B) are shown with BCa 95% bootstrapped confidence intervals based on 5000 stratified resamples by group (fixed random seed). R² change reflects the interaction block. Group-specific slopes represent simple slopes estimated by refitting the model with each group as the reference. Benjamini–Hochberg FDR correction was applied within each variable across the five IPASE subscales: interaction-term p-values were adjusted separately for each contrast (subscale×ASD; subscale×CC; 5 tests each), and group-specific slope p-values were adjusted within each group (CHR/ASD/CC; 5 tests per group).

CHR-P, clinical high risk for psychosis; ASD, autism spectrum disorder; CC, community controls; IPASE, the inventory for psychotic like anomalous self experiences; DACOBS, davos assessment of cognitive biases scale; TASIT-S, the awareness of social inference test - short form; PSP, personal and social performance scale; SSPA, social skills performance assessment.

* Represents significance after Benjamini-Hochberg false discovery rate correction for multiple testing

**Appendix C**. Supplementary Group Comparisons and Associations

**Table C.1** *ANCOVA post hoc multiple comparisons adjusting for sex, age and estimated IQ*

| **Variables** | **Comparison** | **ΔM** | **95% CI** | ***p* (FDR corrected)** |
| --- | --- | --- | --- | --- |
| IPASE |  |  |  |  |
| Total score | CHR-P vs ASD | 42.588 | 24.488, 60.688 | ***p* < .001*** |
|  | CHR-P vs CC | 84.193 | 65.973, 102.414 | ***p* < .001*** |
|  | ASD vs CC | 41.605 | 21.980, 61.231 | ***p* < .001*** |
| Cognition | CHR-P vs ASD | 3.065 | 0.876, 5.255 | ***p* = .007*** |
|  | CHR-P vs CC | 6.041 | 3.837, 8.245 | ***p* < .001*** |
|  | ASD vs CC | 2.976 | 0.602, 5.350 | ***p* = .015*** |
| Self-awareness and presence | CHR-P vs ASD | 21.522 | 13.513, 29.531 | ***p* < .001*** |
|  | CHR-P vs CC | 38.563 | 30.501, 46.626 | ***p* < .001*** |
|  | ASD vs CC | 17.041 | 8.357, 25.725 | ***p* = .013*** |
| Consciousness | CHR-P vs ASD | 3.635 | 0.770, 6.500 | ***p* < .001*** |
|  | CHR-P vs CC | 10.545 | 7.661, 13.429 | ***p* < .001*** |
|  | ASD vs CC | 6.910 | 3.804, 10.016 | ***p* < .001*** |
| Somatization | CHR-P vs ASD | 10.180 | 4.517, 15.843 | ***p* < .001*** |
|  | CHR-P vs CC | 22.251 | 16.550, 27.953 | ***p* < .001*** |
|  | ASD vs CC | 12.072 | 5.931, 18.212 | ***p* < .001*** |
| Demarcation/ Transitivism | CHR-P vs ASD | 4.186 | 2.485, 5.887 | ***p* < .001*** |
|  | CHR-P vs CC | 6.793 | 5.080, 8.505 | ***p* < .001*** |
|  | ASD vs CC | 2.607 | 0.762, 4.451 | ***p* = .006*** |
| DACOBS | CHR-P vs ASD | -0.065 | -12.540, 12.410 | *p* = .992 |
|  | CHR-P vs CC | 47.618 | 35.121, 60.115 | ***p* < .001*** |
|  | ASD vs CC | 47.683 | 34.153, 61.213 | ***p* < .001*** |
| TASIT-S | CHR-P vs ASD | -0.177 | -1.892, 1.538 | *p* = .838 |
|  | CHR-P vs CC | -1.061 | -2.787, 0.666 | *p* = .226 |
|  | ASD vs CC | -0.884 | -2.743, 0.976 | *p* = .348 |
| SSPA | CHR-P vs ASD | 8.372 | 4.372, 12.371 | ***p* < .001*** |
|  | CHR-P vs CC | -7.930 | -11.936, -3.923 | ***p* < .001*** |
|  | ASD vs CC | -16.301 | -20.639, -11.964 | ***p* < .001*** |
| PSP | CHR-P vs ASD | -0.567 | -7.344, 6.210 | *p* = .868 |
|  | CHR-P vs CC | -35.042 | -41.865, -28.220 | ***p* < .001*** |
|  | ASD vs CC | -34.476 | -41.824, -27.127 | ***p* < .001*** |

***Note.*** ANCOVAs adjusted for age, estimated IQ, and sex assigned at birth. Pairwise comparisons were conducted using estimated marginal means. To control for multiple comparisons, p-values from were evaluated using the Benjamini-Hochberg false discovery rate procedure (q = 0.05). Correction was applied within each set of post hoc comparisons.

CHR-P, clinical high risk for psychosis; ASD, autism spectrum disorder; CC, community controls; IPASE, the inventory for psychotic like anomalous self experiences; DACOBS, davos assessment of cognitive biases scale; TASIT-S, the awareness of social inference test - short form; PSP, personal and social performance scale; SSPA, social skills performance assessment.

* Represents significance after Benjamini-Hochberg false discovery rate correction for multiple testing.

**Table C.2** *Unadjusted ANOVA differences between groups on level of self-disturbance, social cognition, social functioning (complete-case analysis)*

| **Variables** | **CHR-P (n = 39)**  **Mean (SD)** | **ASD (n = 39)**  **Mean (SD)** | **CC (n = 30)**  **Mean (SD)** | **F and P-value** | **Post hoc FDR corrected** |
| --- | --- | --- | --- | --- | --- |
| IPASE |  |  |  |  |  |
| Total score | 154.6 (36.4) | 110.9 (43.7) | 73.8 (17.7) | F = 44.837, ***p* < 0.001*** | CHR>ASD>CC |
| Cognition | 14.2 (4.9) | 10.7 (4.5) | 8.5 (2.4) | F = 16.350, ***p* < 0.001*** | CHR>ASD>CC |
| Self-awareness and presence | 64.5 (16.5) | 43.0 (19.9) | 27.5 (7.3) | F = 46.875, ***p* < 0.001*** | CHR>ASD>CC |
| Consciousness | 19.2 (5.8) | 14.8 (6.7 | 9.2 (3.3) | F = 27.129, ***p* < 0.001*** | CHR>ASD>CC |
| Somatization | 43.9 (11.4) | 33.3 (13.4) | 22.4 (6.2) | F = 32.168, ***p* < 0.001*** | CHR>ASD>CC |
| Transitivism | 12.82 (3.9) | 9.1 (3.6) | 6.2 (1.5) | F = 35.101, ***p* < 0.001*** | CHR>ASD>CC |
| DACOBS |  |  |  |  |  |
| Total score | 150.1 (23.7) | 147.1 (26.8) | 104.1 (19.7) | F = 37.660, ***p* < 0.001*** | CHR/ASD>CC |
| Jumping to conclusions bias | 20.6 (5.2) | 20.8 (5.8) | 20.9 (5.3) | F = 0.981, ***p* = 0.019*** | n.s. |
| Belief inflexibility bias | 17.7 (4.1) | 17.8 (4.1) | 13.2 (4.3) | F = 13.169, ***p* < 0.001*** | CHR/ASD>CC |
| Attention for threat bias | 24.7 (5.9) | 24.6 (6.2) | 18.2 (6.4) | F = 11.926, ***p* < 0.001*** | CHR/ASD>CC |
| External attribution bias | 18.9 (4.9) | 17.6 (5.4 | 13.3 (3.0) | F = 12.844, ***p* < 0.001*** | CHR/ASD>CC |
| Social cognition | 23.3 (21.5) | 25.9 (5.9) | 13.3 (4.5) | F = 41.199, ***p* < 0.001*** | CHR/ASD>CC |
| Subjective cognitive problems | 29.2 (5.6) | 24.7 (6.5) | 16.3 (4.2) | F = 45.602, ***p* < 0.001*** | CHR>ASD>CC |
| Safety behavior | 15.5 (5.7) | 15.6 (6.9) | 8.8 (2.9) | F = 15.362, ***p* < 0.001*** | CHR/ASD>CC |
| TASIT-S |  |  |  |  |  |
| Total score | 28.9 (3.5) | 29.2 (3.2) | 29.8 (3.4) | F = 0.678, *p =* 0.510 | - |
| Sincere | 12.4 (2.6) | 11.7 (3.5) | 13.2 (3.2) | F = 1.977, *p =* 0.144 | - |
| Simple sarcasm | 8.6 (2.4) | 9.6 (2.3) | 8.9 (2.4) | F = 1.777, *p =* 0.174 | - |
| Paradoxal sarcasm | 7.8 (0.7) | 7.9 (0.4) | 7.7 (0.9) | F = 0.615, *p =* 0.543 | - |
| SSPA |  |  |  |  |  |
| Total score | 66.6 (8.4) | 59.7 (9.1) | 74.6 (3.9) | F = 31.256, ***p* < 0.001*** | CC>CHR>ASD |
| Subscore: Scene 1 | 32.4 (5.4) | 27.3 (5.4) | 35.9 (2.8) | F =28.030, ***p* < 0.001*** | CC>CHR>ASD |
| Subscore: Scene 2 | 34.3 (3.9) | 32.5 (5.3) | 38.7 (2.1) | F = 19.924, ***p* < 0.001*** | CHR/ASD>CC |
| PSP | 54.2 (11.7) | 54.2 (17.6) | 89.1 (6.5) | F = 76.333, ***p* < 0.001*** | CHR/ASD>CC |

***Note***. Group differences were tested with one-way ANOVA using raw scale scores for all measures. To control for multiple testing, p-values were evaluated using the Benjamini–Hochberg false discovery rate (FDR) procedure with q = .05. Post hoc pairwise comparisons were conducted using Games–Howell; “>” indicates a statistically higher score (p < .05). “/” indicates no significant difference. n.s. = no significant pairwise differences.

CHR-P, clinical high risk for psychosis; ASD, autism spectrum disorder; CC, community controls; IPASE, the inventory for psychotic like anomalous self experiences; DACOBS, davos assessment of cognitive biases scale; TASIT-S, the awareness of social inference test - short form; PSP, personal and social performance scale; SSPA, social skills performance assessment.

* Represents significance after Benjamini-Hochberg false discovery rate correction for multiple testing

**Figure C.3** *Unadjusted associations between anomalous self-experiences and DACOBS across groups* *(complete-case analysis)*

***Note.*** Scatterplot showing the relationship between IPASE total score and DACOBS total score in CC, individuals with ASD, and those at CHR-P. While CHR-P participants showed the highest overall levels of both self-disturbance and attributional bias, the strongest association between the two was observed in the ASD group (R² = 0.396), with weaker associations in CC (R² = 0.125) and CHR (R² = 0.074).

CHR-P, clinical high risk for psychosis; ASD, autism spectrum disorder; CC, community controls; IPASE, the inventory for psychotic like anomalous self experiences; DACOBS, davos assessment of cognitive biases scale.

**Figure C.4** *Unadjusted associations between anomalous self-experiences and social functioning across groups (complete-case analysis)*

***Note.*** Scatterplot illustrating the association between IPASE total score and social functioning (PSP total score). Negative associations were observed in the ASD (R² = 0.110) and CC (R² = 0.134) groups, while no relationship was evident in the CHR-P group (R² ≈ 0). CHR-P, clinical high risk for psychosis; ASD, autism spectrum disorder; CC, community controls; IPASE, the inventory for psychotic like anomalous self experiences; PSP, personal and social performance scale.

**Appendix D**. Correlation Matrices

***
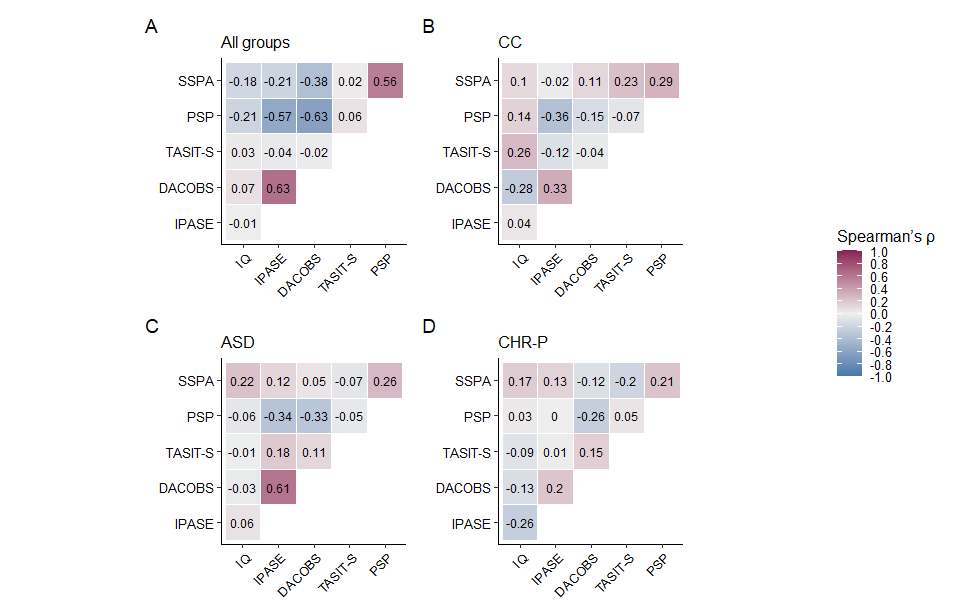
*Figure D.1** *Spearman correlation matrices among IPASE and social cognitive/social functioning variables by group*

***Note.*** Heatmaps display Spearman’s rank correlations (ρ) among estimated IQ, IPASE total score, DACOBS, TASIT-S, PSP, and SSPA for the full sample and within each group (CC, ASD, CHR-P). Values reflect pairwise complete observations. Color intensity indicates the magnitude and direction of the correlation (negative to positive), and coefficients are shown in the upper triangle.

CHR-P, clinical high risk for psychosis; ASD, autism spectrum disorder; CC, community controls; IQ, estimated full-scale IQ (WAIS-IV two-subtest form); IPASE, the inventory for psychotic like anomalous self-experiences; DACOBS, davos assessment of cognitive biases scale; TASIT-S, the awareness of social inference test - short form; PSP, personal and social performance scale; SSPA, social skills performance assessment.
